# A Comparison of Coupling Strategies for 1D–3D Simulations of Coronary Hemodynamics

**DOI:** 10.1002/cnm.70126

**Published:** 2025-12-10

**Authors:** P. L. J. Hilhorst, A. J. E. Vermeer, K. Zając, M. van 't Veer, M. Rezaeimoghaddam, F. N. van de Vosse, W. Huberts

**Affiliations:** ^1^ Department of Biomedical Engineering Eindhoven University of Technology Eindhoven the Netherlands; ^2^ Department of Cardiology Catharina Hospital Eindhoven the Netherlands; ^3^ Sano Centre for Computational Medicine Kraków Poland; ^4^ Computational Science Lab, Faculty of Science, Institute for Informatics University of Amsterdam Amsterdam the Netherlands

**Keywords:** computational fluid dynamics, coronary hemodynamics, patient‐specific modeling, pulse‐wave propagation model, reduced‐order modeling

## Abstract

Noninvasive prediction of Fractional Flow Reserve (FFR) from imaging data through computational modeling has emerged as a promising alternative to invasive pressure measurements. Simulating coronary physiology, specifically coronary stenosis, poses a significant challenge due to the complex geometries of stenotic lesions and the need for physiologically realistic boundary conditions. Coupled 1D–3D modeling frameworks integrate a global one‐dimensional (1D) circulation model with localized three‐dimensional (3D) Computational Fluid Dynamics (CFD), enabling dynamic updates of boundary conditions and more accurate hemodynamic simulation. In this study, we couple a global 1D model of the coronary tree and partial systemic circulation with 3D CFD simulations using synthetically generated coronary stenosis geometries. We created three lesion types—symmetric, eccentric, and irregular—at severities of 50%, 70%, and 80%, to evaluate explicit coupling strategies for FFR prediction. We compare a steady‐state 3D simulation driven by mean flow from the transient 1D model with transient 3D simulations that exchange data continuously at every step or only at the end of the converged cardiac cycle. Applied to the synthetic stenotic geometries, all approaches predicted similar FFR values, while the steady‐state strategy achieved a significant reduction in computational cost, rendering it the most efficient for FFR prediction. Moreover, for irregular lesion geometries, localized 3D modeling revealed discrepancies in pressure loss compared to a simplified lumped model, demonstrating the added value of high‐fidelity 3D simulations in complex cases.

## Graphical abstract

Various 1D–3D coupling strategies combining a one‐dimensional (1D) pulse wave propagation model with three‐dimensional (3D) computational fluid dynamics (CFD) were evaluated for numerous types of complex synthetic coronary lesion geometries. Steady‐state simulations driven by mean flow showed no discrepancy with transient simulations in predicting Fractional Flow Reserve (FFR) while substantially reducing computational cost.

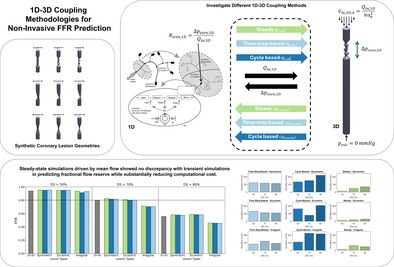

## 1 Introduction

Coronary physiology plays a crucial role in guiding revascularization strategies for patients with coronary artery disease [1]. Traditionally, physiology is assessed invasively using intracoronary pressure wires, in combination with pharmacological agents to induce hyperemia. A key index in this context is the Fractional Flow Reserve (FFR), which quantifies the physiological severity of coronary lesions by measuring the pressure distal to the stenosis and at the aortic root during hyperemia [2]. The ratio of these pressures reflects the reduction in blood flow caused by the stenosis relative to a healthy vessel. In clinical practice, the FFR is used to defer revascularisation when its value is above 0.80. Despite its diagnostic value, the routine use of FFR remains limited due to increased procedural time and potential patient discomfort from hyperemia‐inducing medications [3].

Image‐based assessment of FFR has emerged as a promising alternative to invasive pressure measurements, eliminating the need for pressure wires and hyperemic agents [4]. These methods leverage anatomical information from coronary angiography, computed tomography (CT), or intravascular imaging, combined with computational models of blood flow [3, 5]. Early image‐based FFR methods relied on complex three‐dimensional (3D) computational fluid dynamics (CFD) computations with detailed transient boundary conditions to derive coronary pressure losses [6, 7]. These models were later simplified either by adopting steady boundary conditions [8], or by transforming them into reduced‐order or lumped models that estimate pressure loss using Poiseuille and Bernoulli‐like laws, with geometry parameters and flow as inputs [9, 10]. Although these simplifications significantly reduce computational time, they do not fully capture the complex anatomical and flow characteristics of coronary lesions [10, 11]. Moreover, the accuracy of these models strongly depends on boundary conditions, particularly the estimation of hyperemic coronary flow, which is often derived from scaling laws or population‐averaged assumptions [3].

An alternative strategy is the coupled 1D–3D modeling framework introduced by Formaggia et al. [12], that enables dynamic interaction between a detailed model of a complex vascular geometry and a simple model of the remaining arterial system. Their approach allows a bidirectional exchange of information: the one‐dimensional (1D) model provides boundary conditions to a localized 3D model, while the 3D model returns detailed flow and pressure information that informs the global circulation [12]. Quarteroni et al. [13] further established the well‐posedness of such an approach. These 1D–3D frameworks have been successfully applied to models of carotid bifurcations [14, 15, 16, 17] or aneurysms [18, 19], using primarily 3D fluid–structure interaction (FSI) techniques. Notably, Passerini et al. [16] introduced a lumped 0D model between the 1D model and 3D CFD computation to account for the rigidity of the vessel in the 3D simulation, while other studies incorporated compliance directly into the 3D model via FSI.

In the context of coronary hemodynamics, Grande Gutiérrez et al. [11] introduced the first 1D–3D coupling method for the coronary circulation, demonstrating comparable accuracy to full 3D simulations in terms of global flow distribution, FFR, and local wall shear stress with significant reduction of computational time. Nevertheless, simulations still required between 30 and 100 h, underscoring the need for more computationally efficient coupling strategies.

While these efforts underscore the promise of 1D–3D coupling, important questions remain regarding how best to integrate the 3D component, particularly in balancing accuracy with computational efficiency. In this work, we propose and evaluate three explicit strategies for integrating a localized 3D CFD model into a global 1D representation of the coronary circulation to simulate hemodynamics around a stenosis. The global 1D model encompasses the coronary arteries and a simplified systemic circulation, while the 3D CFD model is embedded at the site of a stenosis to resolve detailed local flow and pressure dynamics. Each coupling strategy differs in how information is exchanged between the 1D and 3D domains. By leveraging these hybrid 1D–3D approaches, we aim to enhance the estimation of boundary conditions for 3D simulations and enable detailed local analysis of stenotic fluid dynamics without incurring the computational cost of a fully 3D model. Importantly, the 1D–3D framework allows for the inclusion of complete lesion geometry in the 3D domain, enabling more anatomically accurate and physiologically relevant simulations. The proposed strategies are applied to synthetic stenotic geometries to assess their accuracy in computing FFR, with comparison against a reduced‐order lumped model [9].

## 2 Methods

**FIGURE 1:**
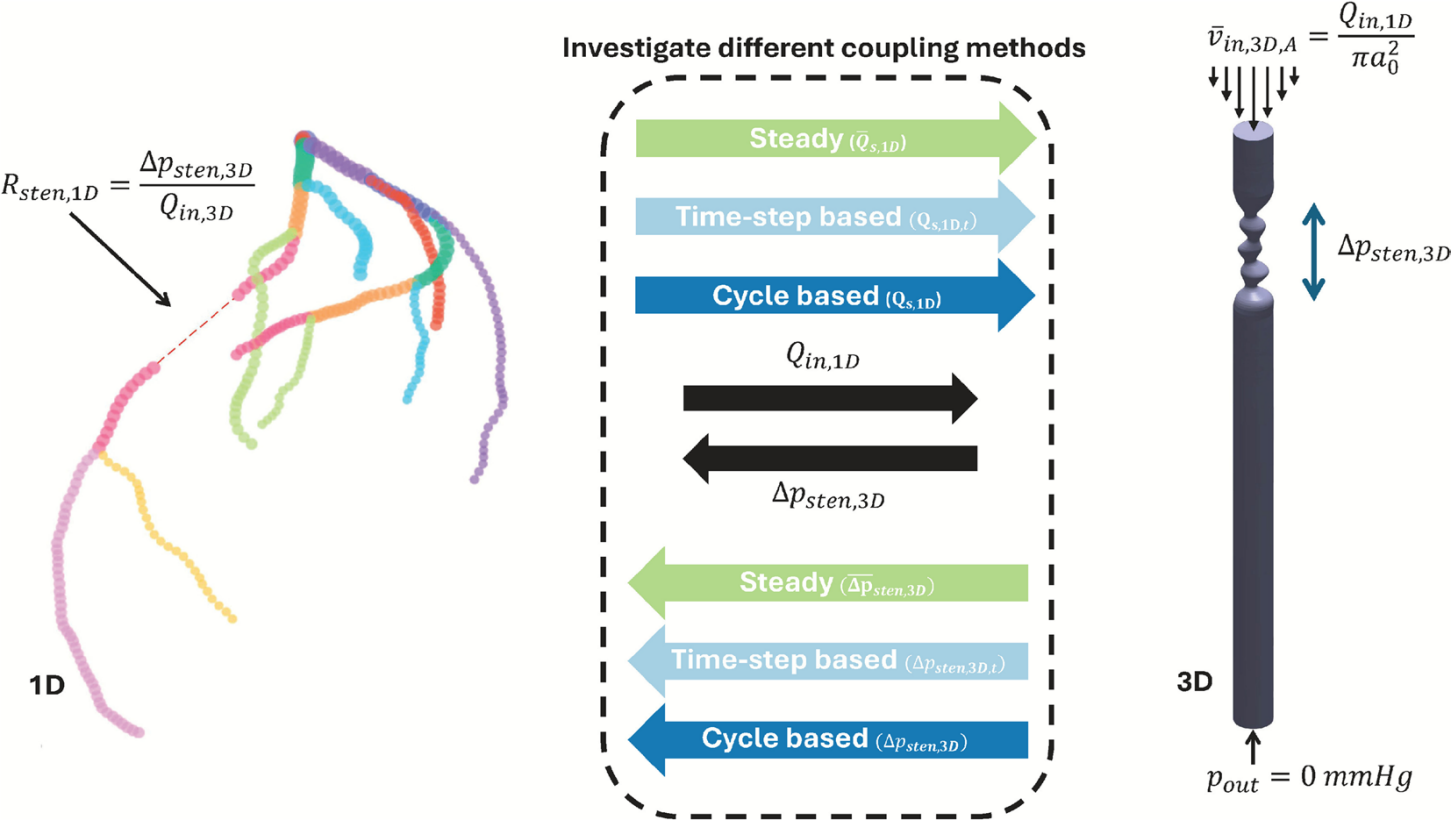
An overview of the explicit 1D–3D coupling approaches described in this paper. Please note that $Q_{\text{in},3D}$ is equal to $Q_{\text{in},1D}$.

This Methods section describes the multi‐scale 1D–3D modeling framework, outlined in Figure 1. The framework couples a 1D pulse wave propagation model of the coronary and systemic circulation with a localized 3D CFD lesion model through an explicit information exchange protocol. Specifically, the flow predicted by the 1D model upstream of the stenosis is imposed as a parabolic velocity profile at the inlet of the 3D domain. The resulting pressure drop, computed from the 3D simulation, is corrected for Poiseuille losses in the geometric extensions to isolate the lesion‐specific contribution. This isolated pressure drop $\Delta p_{\text{sten},3D}$ is translated into an equivalent resistance, which is prescribed back into the 1D model.

The remainder of this section is organized as follows. First, we present a concise overview of the 1D pulse‐wave propagation model, which incorporates a zero‐dimensional (0D) one‐fiber heart model and the 0D lumped stenosis element of Heinen et al. [9]. The latter is used both to initialize the hybrid simulations and to serve as a comparison with the 1D–3D coupling results. We then describe the setup of the 3D CFD lesion model, including geometry generation, meshing, and solver configuration. Next, we introduce the explicit coupling schemes devised to exchange flow and pressure information between the 1D and 3D domains. Three distinct approaches were implemented: time‐step‐based coupling, cycle‐based coupling, and steady coupling. In the time‐step‐based approach, flow and pressure drop are exchanged between the 1D and 3D models at every time step of the cardiac cycle. The cycle‐based approach instead performs this exchange after a complete cardiac cycle in the 1D model, using the resulting transient flow profile as input for the 3D simulation. The steady coupling method follows a similar setup to the cycle‐based approach but simplifies the 3D simulation by using the mean flow from the 1D model as inlet condition. The 3D model then computes a steady‐state pressure drop, which is used to update the 1D model. An overview of simulations and description of the computational infrastructure are provided in the final parts of this section.

### 2.1 1D Pulse Wave Propagation Model

We employ the one‐dimensional pulse wave propagation model described in our previous study by Hilhorst et al. [20]. In this model, the systemic and coronary vessels are represented as 1D line elements governed by the conservation equations for mass and momentum. To enhance computational efficiency, the systemic arterial tree is truncated distal to the region of interest, with the peripheral vasculature beyond the truncation points described using three‐element Windkessel models, referred to as systemic Windkessels. The systemic arteries primarily consist of the aortic arch and a few major branching vessels originating from it, capturing the essential hemodynamic behavior while simplifying the peripheral circulation. In contrast to our original implementation, the arterial wall mechanics are now described using the material law proposed by van der Horst et al. [21], providing a more physiologically accurate representation of arterial compliance. The coronary tree connects to the systemic tree at the aortic root, which is the origin of the systemic domain. The coronary microcirculation is represented using specialized lumped parameter constructs, termed coronary Windkessels. Hyperemia is modeled by reducing the total resistance of the coronary microcirculation by a fixed factor. In contrast to the original setup, this factor is set to 3, corresponding to a threefold reduction in resistance, consistent with clinical observations [22]. In the stand‐alone 1D model, stenotic lesions are described by the nonlinear lumped resistance element of Heinen et al. [9]. The heart is modeled using a 0D one‐fiber heart model, which provides the inflow boundary condition at the root of the systemic tree. This heart model is coupled to the coronary windkessels to capture the effects of intramyocardial pressure on coronary artery dynamics. For a comprehensive description of the governing equations and model formulation, we refer the reader to Hilhorst et al. [20].

#### 2.1.1 Coronary Topology

**FIGURE 2:**
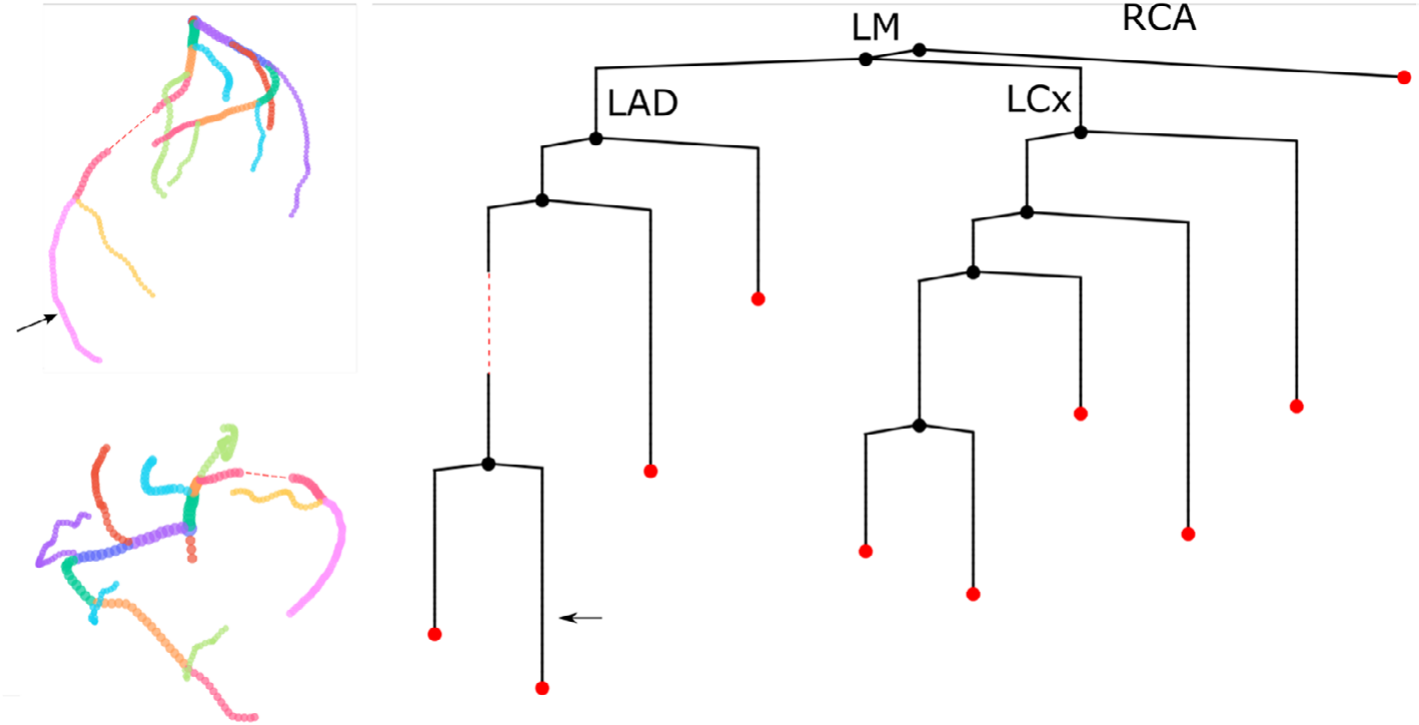
A 3D representation (left) and schematic representation (right) of the 1D coronary topology derived from segmented angiography data. In the 3D representation, each vessel segment is represented with a unique color. The red striped line indicates the location where the stenosis occurs. The black arrow indicates the measurement location of the distal pressure for computation of the FFR. The main epicardial arteries, which are the Left Main (LM), Left Anterior Descending (LAD), Left Circumflex (LCx), and Right Coronary Artery (RCA), are denoted. Although the LM and RCA are not directly attached, they share a common origin on the aorta, hence the schematic depiction of them branching from the same vessel.

The coronary geometry in the 1D model was derived from coronary angiography data acquired in the FAME 2 study [23]. A 3D reconstruction using Pie Medical Imaging CAAS provided the centerline coordinates and radii for the left coronary artery (LCA). As no reconstruction of the right coronary artery (RCA) was available, a 1D RCA segment (radius 1.59 mm, length 3.19 mm), was manually added to ensure physiologically consistent flow distribution between the left and right coronary systems. The complete coronary topology can be seen in Figure 2. Wall thickness of all coronary vessels was assumed to be 10% of the vessel's radius [21]. For a detailed description of the systemic artery topology and how it was truncated, the reader is referred to Hilhorst et al. [20].

#### 2.1.2 Lumped Stenosis Model

The original setup of the 1D–0D model described coronary stenoses using the geometry‐based model by Heinen et al. [9]. This model establishes a relation between pressure drop (Δp) and flow (Q) over an idealized, symmetric stenotic lesion, based on typical stenosis characteristics, such as the minimal radius ($a_{s}$) and lesion length ($l_{s}$). The lumped stenosis element can be represented by

(1) $$\Delta p=K_{v}\;\underset{\text{Viscous term}}{\underbrace{\frac{8\mu l_{s}}{\pi a_{0}^{4}}\;Q}}+K_{t}\;\underset{\text{Inertial term}}{\underbrace{\frac{\rho }{2\pi^{2}a_{0}^{4}}{\left[{\left(\frac{a_{0}}{a_{s}}\right)}^{2}-1\right]}^{2}Q^{2}}}$$

The first term represents the pressure drop due to viscous forces, whilst the second term represents the pressure drop due to inertial forces. Here μ is the dynamic viscosity, $l_{s}$ is the length of the stenotic lesion, $a_{0}$ is the radius of a healthy vessel, $a_{s}$ is the minimal stenosis radius, $K_{v}$ and $K_{t}$ are the geometry‐dependent dimensionless viscous and inertial loss coefficients, respectively. For a more elaborate description, we refer the reader to our previous work [9].

#### 2.1.3 Boundary Conditions

**FIGURE 3:**
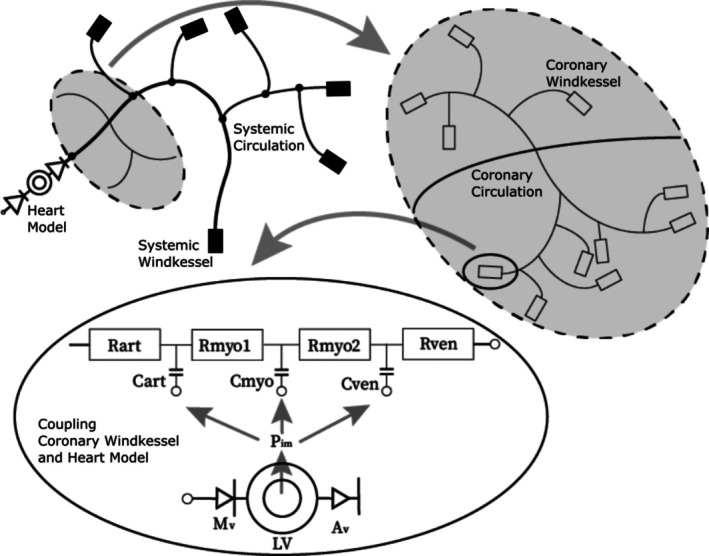
A schematic overview of the 1D pulse wave propagation model consisting of a small part of the systemic circulation, the coronary circulation, and lumped models serving as boundary conditions. Taken from [20]. *Rart*, *Rmyo1*, *Rmyo2* and *Rven* are the resistance parameters, *Cart*, *Cmyo* and *Cven* are the compliance values and *Pim* is the intramyocardial pressure within the coronary Windkessel model. Here *art* stands for arteriolar, *myo* for myocardial, *ven* for venular, *Mv* for mitral valve, *LV* for left ventricle, and *Av* for aortic valve.

At the inlet of the 1D model, a one‐fiber heart model, explained in [20], is used to derive a time‐dependent inflow into the systemic circulation. In this model, a constant pulmonary venous pressure of $P_{\mathrm{pul}\;\mathrm{ven}}$ = 1067 Pa is prescribed as preload [21]. The external resistance nodes in the Windkessel elements have a venous outlet pressure of $P_{\mathrm{vo}}$ = 700 Pa [24]. In the systemic windkessels, the external pressure on the compliance nodes was set to 0 Pa. In the coronary windkessel models, the effect of myocardial contraction ($P_{\mathrm{im}}$) is considered and prescribed on the compliance nodes, as visible in Figure 3. A more elaborate description of how intramyocardial pressure is incorporated, is provided in our previous work [20].

#### 2.1.4 Numerical Implementation

The 1D–0D model was implemented in Python 3.9. The solution method leverages the finite element‐based numerical method of reduced complexity described by Kroon et al. [25]. To ensure numerical convergence and minimize truncation error, the 1D systemic and coronary artery segments were discretized with element lengths of $\Delta z_{\mathrm{sys}}=10$ mm and $\Delta z_{\mathrm{cor}}=1$ mm, respectively. Each element consists of two nodes with pressure and flow as degrees of freedom, ensuring continuity through shared nodes. Flow is defined inwards at each node and the Trapezoidal rule is applied for spatial integration of the 1D mass and momentum equations. Time discretization is performed using a second‐order backward‐difference scheme with a time step of $\Delta t=1.8\times 10^{-3}\;s$. A cardiac cycle consisted of 500 time steps, corresponding to a heart rate of 65 bpm.

### 2.2 3D Computational Fluid Dynamics Model

**FIGURE 4:**
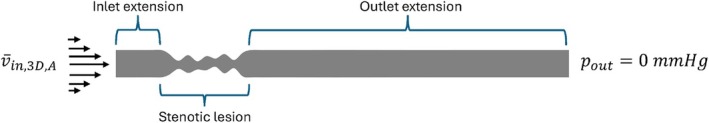
An overview of the computational fluid dynamics setup in ANSYS Fluent.

A schematic overview of the 3D CFD analysis setup is provided in Figure 4.

The 3D geometry consists of three regions: a straight inlet extension (5 mm), a synthetic stenotic lesion segment (10 mm), and a straight outlet extension consisting of the original geometry (5 mm) and an added extension equal to 10 times the vessel diameter (30 mm). Hence the total length of the extensions is $l_{\mathrm{ext}}=40\;\mathrm{mm}$.

At the inlet, a parabolic velocity profile with an area‐averaged (indicated by subscript A) velocity ${\bar{v}}_{\text{in},3D,A}$ is prescribed, corresponding to the 1D flow rate $Q_{\text{in},1D}$. A parabolic velocity profile was selected based on the inlet Reynolds numbers under hyperemic flow conditions (using the mean flows in Table 1) which remain below 500 (well within the laminar regime with threshold = 2000) and a Womersley number (α = 2.15) extracted from the 1D pulse wave propagation model, indicating quasi‐steady, near parabolic flow even if not fully developed. In addition, prescribing the inlet velocity (rather than the pressure drop) is essential in our 1D–3D coupling framework, since the pressure drop across the lesion is the output of interest and is intended to be predicted by the 3D solver, not imposed. This way, the pressure loss emerges naturally from the computed flow field, ensuring consistency between the models.

**TABLE 1:** Mean coronary flow per main epicardial artery under hyperemic conditions.

Artery	Flow (mL/min)
LAD	222
LCx	211
RCA	193

The area‐averaged inlet velocity is computed from the 1D result by dividing the flow rate by the inlet cross‐sectional area (${\bar{v}}_{\text{in},3D,A}=\frac{Q_{\text{in},1D}}{\pi \;a_{0}^{2}}$). This ${\bar{v}}_{\text{in},3D,A}$ is then used to construct a parabolic profile, that is prescribed in the z‐direction as:

(2) $$v_{\text{in},3D}\left(x,y\right)\;=\;2\;{\bar{v}}_{\text{in},3D,A}\left(t\right)\;\left(1\;-\;\frac{x^{2}+y^{2}}{a_{0}^{2}}\right)$$

We acknowledge that while in vivo coronary inflows may be flatter or more plug‐like, the parabolic assumption is consistent with the laminar regime at the inlet and with the 1D model formulation. This choice ensures a smooth and stable boundary condition while keeping the coupling procedure tractable.

At the outlet of the domain a fixed reference pressure $p_{\text{out}}=0\;\text{mmHg}$ is prescribed. To isolate the stenotic contribution to the pressure drop, we do not measure pressures immediately upstream and downstream of the lesion, since such local measurements can be strongly influenced by flow separation, recirculation zones, and secondary motions, which makes the definition of a representative Δp ambiguous. Instead, the theoretical viscous losses in the straight, non‐stenotic extensions are computed via Poiseuille's law and subtracted from the total inlet–outlet pressure drop. We acknowledge that, strictly speaking, Poiseuille's assumption of laminar, fully developed flow may not hold distal to a stenosis. However, additional transient simulations (not included in the current manuscript) showed that the discrepancy between this subtraction method and a direct lesion‐to‐lesion measurement was small, with a maximal difference below 2 mmHg. This confirms that the subtraction approach provides a simple, robust, and reproducible estimate of the stenosis‐induced pressure loss consistently across all simulated geometries.

Hence, to remove the baseline viscous losses in the straight, non‐stenotic extensions and isolate the pressure drop attributable solely to the stenotic lesion, we first compute.

(3) $$\Delta p_{\text{Poiseuille}}=\frac{8\;\mu \;l_{\mathrm{ext}}\;Q_{\text{in},1D}}{\pi \;a_{0}^{4}}$$

and then define the stenotic pressure drop in the 3D model as

(4) $$\Delta p_{\text{sten},3D}={\bar{p}}_{\text{in},A}-{\bar{p}}_{\text{out,A}}-\Delta p_{\text{Poiseuille}}={\bar{p}}_{\text{in},A}-\frac{8\;\mu \;l_{\mathrm{ext}}\;Q_{\text{in},1D}}{\pi \;a_{0}^{4}}$$

with ${\bar{p}}_{\text{in},A}$ and ${\bar{p}}_{\text{out},A}$ as the area‐averaged pressures at the inlet and outlet, respectively.

Now that we have provided the generic CFD setup, we will first describe the generation of synthetic coronary lesions; next, elaborate on the volumetric meshing; and finally, outline the detailed setup of the fluid dynamics solver.

#### 2.2.1 Generation of Synthetic Coronary Lesions

**FIGURE 5:**
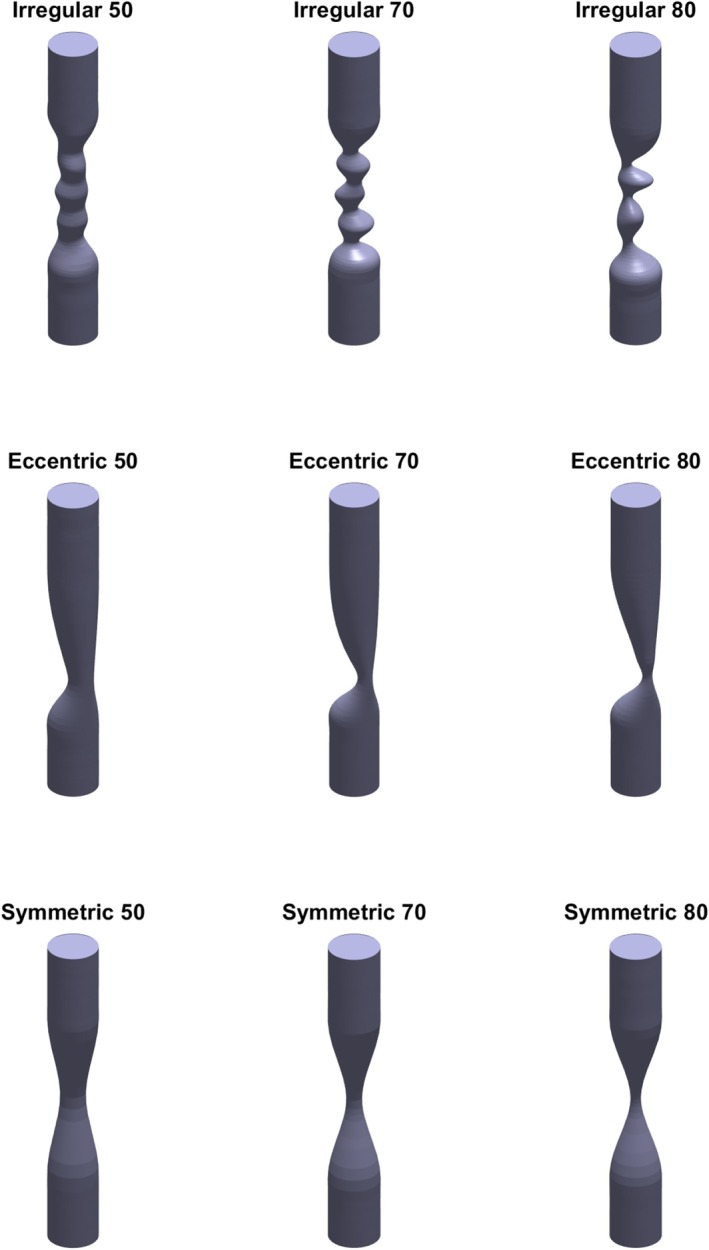
An overview of the 3D stenotis geometries used for the 3D CFD simulations, the 30 mm extensions are not shown here. Three different lesion types were defined: irregular (top row), eccentric (middle row), and symmetric (bottom row). Diameter severity was varied between 50% (left column), 70% (middle column) and 80% (right column).

Synthetic geometries of coronary stenoses were generated using SolidWorks (2023, SolidWorks, Dassault Systèmes). Each geometry initially comprises a 10 mm lesion flanked by two 5 mm healthy regions of reference diameter D=3mm. The diameter reduction at the minimum diameter location was 50%, 70%, or 80% with respect to the reference diameter. The radius profiles were varied to create three distinct lesion types: symmetric, eccentric, and irregular. To generate these geometries, 20 equidistant planes (1 mm apart) were defined: the first and last five planes featured 3 mm diameter circles, while the middle 10 planes had the circle radii and shapes modified to produce the lesion profiles. The combination of lesion types with diameter severities results in nine unique synthetic geometries, depicted in Figure 5. To account for the recirculation zones typically observed distal to the lesion, cylindrical flow extensions 10 times the reference diameter (30 mm) in length were attached to the distal end of the domain [26]. Figure 4 shows a 2D view of the irregular geometry with 70% diameter reduction, including the inlet and outlet extensions indicated.

#### 2.2.2 Volumetric Meshing

The geometry files describing the outer surface of the fluid domain were imported into Fluent Meshing 2024R1 (Ansys Inc., Canonsburg, PA, USA) for volumetric meshing. The watertight geometry workflow was utilized to ensure that the geometry was fully enclosed, without any gaps or leaks. Polyhedral cells were chosen for the discretization of the volume domain. Mesh sizes were determined through a grid dependence analysis, which involved successive refinements of the mesh. From these analyses, an element edge length of $8\times 10^{-5}$ m with a growth rate of 1.2 was derived for the surface mesh, meaning each successive element may be at most 1.2 times larger than its neighbor. For the volume mesh, the maximum cell length was set to 0.1 mm, with a similar growth rate of 1.2. The total number of cells across all domains ranged between 383,000 and 460,000 cells. The volumetric meshes were parsed to the CFD solver for simulation.

#### 2.2.3 Fluid Dynamics Solver

In the three‐dimensional domain, the governing equations of fluid flow are the three‐dimensional conservation of mass and the momentum balance, which read in differential form, respectively:

(5) $$\frac{\partial \rho }{\partial t}+\nabla \cdot \left(\rho \vec{v}\right)=0$$

(6) $$\rho \frac{\partial \vec{v}}{\partial t}+\rho \left(\vec{v}\cdot \nabla \right)\vec{v}=\vec{f}+\nabla \cdot \sigma$$

where ρ is the fluid density, t the time, $\vec{v}$ the velocity vector, $\vec{f}$ the body forces per unit volume, σ the Cauchy stress tensor and ∇ the gradient operator. When it is assumed that the fluid is a homogeneous, incompressible material, the fluid density is constant, which implies that $\frac{\mathrm{d\rho}}{\mathrm{dt}}=0$ and ∇⋅ρ=0. Under this assumption the mass balance reduces to the continuity equation:

(7) $$\nabla \cdot \vec{v}=0$$

The Cauchy stress tensor (σ) in the momentum equation can be decomposed into a static pressure term (p) and an extra stress tensor τ, such that

(8) σ=−pI+τ

While assuming an incompressible Newtonian fluid, the density is constant. Additionally, the viscosity is shear‐rate independent, which physically means that the stress tensor τ=2ηD, with $D=\frac{1}{2}\left(\nabla \vec{v}+{\left(\nabla \vec{v}\right)}^{T}\right)$ being the velocity‐gradient tensor. Furthermore, body forces $\vec{f}$ were neglected.

Under these conditions, the momentum balance becomes:

(9) $$\frac{\partial \vec{v}}{\partial t}+\left(\vec{v}\cdot \nabla \right)\vec{v}=-\frac{1}{\rho }\nabla p+\nu \nabla^{2}\vec{v}$$

with $\nu =\frac{\mu }{\rho }$ the kinematic viscosity.

The mass and momentum balance equations were solved in ANSYS Fluent 2024R1 (Ansys Inc., Canonsburg, PA, USA) using a finite‐volume formulation. In this control‐volume‐based technique, the geometry is discretized into control volumes using a grid, creating a volumetric mesh, as described in Section 2.2.2. The integral conservation laws are approximated for each volume through discretization and linearization, constructing a linear system of algebraic equations. A co‐located scheme stores the unknowns (pressure and velocity) in these equations at the cell centers. Convective terms in the momentum equations were discretized using ANSYS Fluent's second‐order upwind scheme, which extrapolates face values from the two upstream cell‐center values with gradient correction and applies slope limiters to maintain boundedness and stability. A least squares cell‐based method was used to estimate the gradients within the equations, and a second‐order scheme for pressure interpolation to faces. Time integration employed a second‐order implicit scheme, and the resulting linear system was iteratively solved using Gauss–Seidel. Pressure–velocity coupling was handled by the SIMPLE algorithm, which first solves the momentum equations to obtain an intermediate velocity field, then solves a pressure‐correction equation to enforce continuity, and finally updates the velocities and pressure in sequence until convergence. For further details on the discretization schemes and solver settings, we refer the reader to the ANSYS Fluent User's Guide [27].

In our simulation setup, laminar blood flow was assumed, with density and dynamic viscosity set to 1060 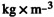 and 0.0035 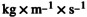, respectively, similar to the settings of the 1D model. The generic inlet and outlet boundary conditions were specified at the beginning of this section. The domain walls were considered rigid, and a no‐slip boundary condition was applied.

Convergence of one time step within the CFD simulation was judged on the sum of the per‐cell equation imbalances (the L_1_‐norm of the residuals), globally scaled by their respective scale factors. The convergence thresholds were set to $10^{-4}$ for the continuity residual and $10^{-5}$ for each velocity‐component residual. The time step size of the 3D model was set to match that of the 1D model, using Δt of 1.8 ms. A time step sensitivity analysis was performed to verify this choice, during which the number of time steps per cardiac cycle was varied, resulting in a smaller time step size with each refinement. Based on this analysis, a total of $N_{\mathrm{ts},1D3D}=500$ time steps per cycle, identical to the 1D model, was selected to simulate a heart rate of 65 bpm. Each time step was solved using a maximum of 200 iterations.

### 2.3 Coupling Strategies 1D–3D

This section describes the strategies used to couple the 3D CFD lesion model with the 1D coronary circulation model: time‐step‐based, cycle‐based and steady.

#### 2.3.1 Transient—Time‐Step‐Based Coupling

The time‐step methodology facilitates the systematic exchange of information between the three‐dimensional CFD model and the one‐dimensional pulse wave propagation model at each discrete time step. An overview of this scheme is shown in Algorithm 1.

ALGORITHM 1 1: Initialize:

2:   1. Setup 3D solver with a generic healthy inflow; run $N_{c,3D}=1$ cycle, with $N_{\mathrm{ts},1D3D}$ time steps per cycle.

3:   2. Setup 1D model (with lumped stenosis by Heinen et al. [9], see Section 2.4); run $N_{c,1D}=10$ cycles, with $N_{\mathrm{ts},1D3D}$ time steps per cycle.

4:   3. Converged ← False

5: **while** not Converged **do**

6:    **Extract 1D flow**: Extract $Q_{\text{in},1D,n_{t}}$ at time step $n_{t}$

7:     Transform the 1D flow to the 3D area‐averaged velocity

$${\bar{v}}_{\text{in},3D,A,n_{t}}=\frac{Q_{\text{in},1D,n_{t}}}{\pi a_{0}^{2}}$$

 and apply under‐relaxation:

$${\bar{v}}_{\text{in},3D,A,n_{t}}\;=\;\alpha \;{\bar{v}}_{\text{in},3D,A,n_{t}}\;+\;\left(1-\alpha \right)\;{\bar{v}}_{\text{in},3D,A,n_{t-1}}$$

8:    **Advance 3D**: Impose ${\bar{v}}_{\text{in},3D,A,n_{t}}$ as a parabolic 3D inlet velocity‐profile boundary condition (see Equation 2).

9:     Solve 3D for one time step $n_{t}$.

10:    **Extract 3D pressure drop**: Extract $\Delta p_{\text{sten},3D,n_{t}}$.

11:     Compute

$$R_{\text{sten},3D,n_{t}}\;=\;\frac{\Delta p_{\text{sten},3D,n_{t}}}{Q_{\text{in},3D,n_{t}}}$$

12:    **Update 1D**: Substitute lumped stenosis with $R_{\text{sten},3D,n_{t}}$ in the 1D model.

13:     Solve 1D for one time step $n_{t}$ to get new $Q_{\text{in},1D,t_{n+1}}$.

14:    After completing $N_{\mathrm{ts},1D3D}$ time steps for this coupling cycle:

15:     Compute stop criteria errors $E_{\Delta p,n_{c}}$ and $E_{Q3D,n_{c}}$ (see Section 2.4).

16:     **If** $N_{c,1D–3D\;\text{coupling}}\geq 20$ **or** $E_{\Delta p,n_{c}}\leq 0.02$ [−] **or** $E_{Q3D,n_{c}}\leq 0.01$ [−] **then** Converged ← True

17: end while

**FIGURE 6:**
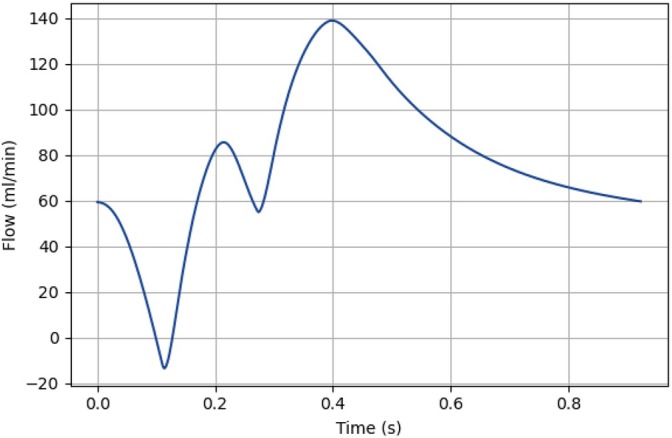
The transient flow waveform that is converted to a parabolic inlet velocity to initialize the 3D CFD model during the time‐step‐based coupling.

First, the 1D pulse wave propagation model is configured, initialized, and executed for 10 cardiac cycles, with the severity percentage of the lumped stenosis element either adjusted to match that of the 3D mesh (by setting the severity of the lumped stenosis element equal to the severity of the corresponding synthetic geometry) or set to 0%, depending on the initialization used (Section 2.4). Subsequently, the 3D CFD model is also configured and initialized. It runs for one cardiac cycle, using a time‐dependent parabolic velocity profile derived from the flow waveform shown in Figure 6 as the inlet boundary condition; this profile is obtained from the 1D pulse wave propagation model representing the healthy proximal stenotic LAD flow.

Once both models have been properly initialized, the flow directly proximal to the stenotic lesion at time step $n_{t}$ ($Q_{\text{in},1D,n_{t}}$) is derived from the 1D pulse wave propagation model. This flow is converted to the inlet area‐averaged velocity (${\bar{v}}_{\text{in},3D,A,n_{t}}$) at time step $n_{t}$. To ensure numerical stability, a 60% under relaxation is applied by taking 60% of the newly computed velocity ${\bar{v}}_{\text{in},3D,A,n_{t}}$ at the current time step ($n_{t}$) and 40% of the velocity ${\bar{v}}_{\text{in},3D,A,t_{n-1}}$ from the previous time step ($n_{t-1}$). The resulting area‐averaged velocity is applied as a parabolic velocity profile, using Equation (2), to the 3D CFD model, which is then solved for one time step. The area‐averaged inlet pressure ${\bar{p}}_{\text{in},A}$ is extracted, and after accounting for the Poiseuille pressure drop (Equation 4), the pressure drop due to the stenosis ($\Delta p_{\text{sten},3D,n_{t}}$) is derived at time step $n_{t}$. The new resistance value $R_{\text{sten},3D,i}$ is obtained by dividing the stenotic pressure drop from the 3D simulation by the corresponding 3D inlet flow. This resistance directly replaces the 0D stenosis element of Heinen et al. [9] and is introduced as a series element between the two 1D nodes delimiting the stenosis, enforcing $\Delta p_{\text{sten}}\left(t\right)=R_{\text{sten},3D,n_{t}}\;Q_{\text{in},1D,n_{t}}$. All other 1D segment parameters and Windkessel elements remain unchanged. The same implementation approach is applied for the other coupling methods in the upcoming sections. Once the resistance value has been updated accordingly, the 1D model is solved for the next time step, resulting in a new $Q_{\text{in},1D,n_{t+1}}$. This process is repeated until convergence is achieved.

#### 2.3.2 Transient—Cycle‐Based Coupling

In the cycle‐based method the 1D and 3D model both run for multiple full cardiac cycles ($N_{c}$) before information is exchanged between the models. The setup of this scheme is described in Algorithm 2.

ALGORITHM 2 **Time Cycle‐Based 1D–3D Coupling.**

1: Initialize:

2:  1. Setup 3D solver

3:  2. Setup 1D model (with lumped stenosis by Heinen et al. [9], see Section 2.4); run $N_{c,1D}=10$ cycles, with $N_{n_{t},1D3D}$ time steps per cycle.

4:  3. Converged ← False

5: **while** not Converged **do**

6:    **Extract 1D flow**: Extract $Q_{\text{in},1D,n_{c}}$ over the last $N_{n_{t},1D3D}$ timesteps, corresponding to one complete cardiac cycle.

7:     Fourier‐transform 1D flow to transient area‐averaged inlet velocity

$${\bar{v}}_{\text{in},3D,A,n_{c}}=\frac{Q_{\text{in},1D,n_{c}}}{\pi a_{0}^{2}}$$

8:    **Advance 3D**: Impose ${\bar{v}}_{\text{in},3D,A,n_{c}}$ as a parabolic 3D inlet velocity profile boundary condition (see Equation 2) using a UDF.

9:     Solve 3D for $N_{c,3D}=2$ cycles.

10:    **Extract 3D pressure drop**: Extract $\Delta p_{\text{sten},3D,n_{c}}$ over the last $N_{n_{t},1D3D}$ timesteps.

11:     Compute

$$R_{\text{sten},3D,n_{c}}\;=\;\Delta p_{\text{sten},3D,n_{c}}⊘Q_{\text{in},3D,n_{c}}$$

where ⊘ denotes element‐wise division.

12:    **Update 1D**: Substitute lumped stenosis with $R_{\text{sten},3D,n_{c}}$ in the 1D model.

13:     Solve 1D for $N_{c,1D}=5$ cycles to get $Q_{\text{in},1D,n_{c+1}}$

14:    After completing 5 cycles for this coupling cycle:

15:     Compute stop criteria errors $E_{\Delta p,n_{c}}$ and $E_{Q3D,n_{c}}$ (see Section 2.3.4).

16:     **If** $N_{c,1D–3D\;\text{coupling}}\geq 20$ **or** $E_{\Delta p,n_{c}}\leq 0.02$ [−] **or** $E_{Q3D,n_{c}}\leq 0.01$ [−] **then** Converged ← True

17: end while

First, the 1D model runs for 10 cardiac cycles ($N_{c,1D}=10$) using the original lumped stenosis element at the site of the lesion to reach hemodynamic equilibrium. The flow waveform from the 10th cycle, $Q_{\text{in},1D,n_{c}}$, is converted into a transient area‐averaged inlet velocity ${\bar{v}}_{\text{in},3D,A,n_{c}}=\frac{Q_{\text{in},1D,n_{c}}}{\pi a_{0}^{2}}$. This profile is fitted with a 20‐harmonic Fourier series and prescribed as a parabolic inlet profile using a user‐defined function (UDF) in ANSYS Fluent.

The 3D model is then run for two cardiac cycles to eliminate initialization effects. From the second cycle, the lesion‐specific pressure loss $\Delta p_{\text{sten},3D,n_{c}}$ is computed using Equation (4). For cycle‐based coupling, we define the time‐resolved resistance vector by the element‐wise division of the pressure drop and flow vectors; $R_{\text{sten},3D,n_{c}}=\Delta p_{\text{sten},3D,n_{c}}\;⊘\;Q_{\text{in},3D,n_{c}}$, that is for each time‐step $n_{t}=1,\ldots ,N_{n_{t},1D3D}$ within the final cardiac cycle c, we compute $R_{\text{sten},3D,n_{c}}\left[n_{t}\right]=\frac{\Delta p_{\text{sten},3D,n_{c}}\left[n_{t}\right]}{Q_{\text{in},3D,n_{c}}\left[n_{t}\right]}$. This time‐resolved resistance vector replaces the lumped stenosis element in the 1D model, with the corresponding resistance value at each corresponding time step $n_{t}$.

Subsequently, the 1D model is run for five additional cycles ($N_{c,1D}=5$) with the new stenotic resistance. If the convergence criteria for $E_{\Delta p,n_{c}}$ and $E_{Q_{3D,n_{c}}}$, as defined in Section 2.3.4, are not met, the coupling loop is repeated. First, two more cardiac cycles of 3D CFD are run to recompute $R_{\text{sten},3D,n_{c}}$ with an updated $Q_{\text{in},1D,n_{c}}$, followed by five cycles of 1D simulations.

#### 2.3.3 Steady Coupling

Whereas the first two coupling methods employ fully transient 3D CFD simulations, this steady‐state coupling approach constrains the 3D solver to a time‐invariant solution. This method is described in Algorithm 3.

ALGORITHM 3 **Steady 1D–3D Coupling.**

1: Initialize:

2:  1. Setup 3D solver

3:  2. Setup 1D model (with lumped stenosis by Heinen et al. [9], see Section 2.4); run $N_{c,1D}=10$ cycles, with $N_{n_{t},1D3D}$ time steps per cycle.

4:  3. Converged ← False

5: **while** not Converged **do**

6:    **Extract 1D flow**: Extract $Q_{\text{in},1D,n_{c}}$ over the last $N_{n_{t},1D3D}$ timesteps.

7:     Compute the area‐ and time‐averaged inlet velocity:

$${\bar{v}}_{\text{in},3D,\mathrm{At},n_{c}}=\frac{1}{N_{n_{t},1D3D}}\sum_{s=1}^{N_{n_{t},1D3D}}\frac{Q_{\text{in},1D,n_{c}}\left(s\right)}{\pi \;a_{0}^{2}}$$

8:    **Advance 3D**: Impose ${\bar{v}}_{\text{in},3D,\mathrm{At},n_{c}}$ as a parabolic 3D inlet velocity profile boundary condition.

9:     Solve 3D for a maximum of 5000 iterations.

10:    **Extract 3D pressure drop**: Extract $\Delta p_{\text{sten},3D,n_{c}}$ at the final iteration.

11:     Compute

$$R_{\text{sten},3D,n_{c}}=\frac{\Delta p_{\text{sten},3D,n_{c}}}{Q_{\text{in},3D,n_{c}}}$$

12:    **Update 1D:** Substitute lumped stenosis with $R_{\text{sten},3D,n_{c}}$ in the 1D model.

13:     Solve 1D for $N_{c,1D}=5$ cycles to get $Q_{\text{in},1D,n_{c}}$ for the last $N_{n_{t},1D3D}$ timesteps.

14:    After completing 5 cycles of the 1D model:

15:     Compute stop criteria errors $E_{\Delta p,n_{c}}$ and $E_{Q3D,n_{c}}$ (see Section 2.3.4).

16:     **If** $N_{c,1D–3D\;\text{coupling}}\geq 20$ **or** $E_{\Delta p,n_{c}}\leq 0.02$ [−] **or** $E_{Q3D,n_{c}}\leq 0.01$ [−] **then** Converged ← True

17: end while

Similarly to the cycle‐based method, the steady coupling starts with running the 1D model for 10 cardiac cycles ($N_{c,1D}=10$) to reach hemodynamic equilibrium. The flow waveform from the 10th cycle $Q_{\text{in},1D,n_{c}}$ is converted into the area‐ and time‐averaged inlet velocity (subscript At) and prescribed as a parabolic velocity profile at the 3D inlet (using Equation 2). A steady 3D simulation is then performed to compute the stenotic pressure drop $\Delta p_{\text{sten},3D,n_{c}}$ (using Equation 4). From this, the corresponding resistance is calculated as $R_{\text{sten},3D,n_{c}}=\frac{\Delta p_{\text{sten},3D,n_{c}}}{Q_{\text{in},3D,n_{c}}}$ and used to replace the lumped stenosis element in 1D. The 1D model continues with five additional cycles ($N_{c,1D}=5$). If the convergence criteria in Section 2.3.4 for $E_{\Delta p,n_{c}}$ and $E_{Q_{3D,n_{c}}}$ are not met, the coupling loop is repeated. In this case, a new steady CFD simulation is performed to recompute $R_{\text{sten},3D,n_{c}}$ using the updated inlet flow $Q_{\text{in},1D,n_{c}}$, followed by five 1D cardiac cycles.

#### 2.3.4 Convergence of Coupling Methods

Convergence of the 1D–3D coupling approaches is based on the comparison of the time‐averaged stenotic pressure loss between the 1D and 3D model. Mathematically, the pressure‐based convergence metric for the coupling approaches can be formulated as

(10) $$E_{\Delta p,n_{c}}=\frac{|{\bar{\Delta p}}_{\text{sten},1D,n_{c}}\;-\;{\bar{\Delta p}}_{\text{sten},3D,n_{c}}|}{{\bar{\Delta p}}_{\text{sten},3D,n_{c}}}$$

where c denotes the cardiac cycle, and ${\bar{\Delta p}}_{\text{sten},3D,n_{c}}$ and ${\bar{\Delta p}}_{\text{sten},1D,n_{c}}$ are the mean pressure drops over the final cycle of the 3D and 1D models, respectively.

In the steady‐state coupling approach, there is no cycle‐to‐cycle variation in the 3D solution, so ${\bar{\Delta p}}_{\text{sten},3D,n_{c}}$ is taken as the single converged pressure drop from the steady 3D CFD simulation. This constant value then serves as the reference denominator in $E_{\Delta p,n_{c}}$. This convergence metric is evaluated immediately after the data exchange between the 1D and 3D solvers in each coupling iteration. In the time‐step‐based coupling, one coupling iteration corresponds to a single time step, so $E_{\Delta p,n_{c}}$ is computed at the end of every time step. In the cycle‐based and steady‐state coupling approaches, a coupling iteration corresponds to a round of multiple cardiac cycles depending on the model dimension (1D or 3D), and the metric is therefore evaluated after the completion of the cycles for the last cycle. Relative pressure differences ($E_{\Delta p}<2\%$) were deemed converged, consistent with reported systemic pressure variations <3% across 1D, 3D, and coupled models [11].

To complement the pressure‐based convergence criterion, we introduce a flow‐based metric. The coupling is terminated when the relative change in mean 3D coronary flow between successive iterations falls below 1%. This threshold also aligns with reported systemic flow variations of under 1% across 1D, 3D, and coupled models [11]. In written form, the flow‐based convergence metric terminates the coupling when the relative change in mean 3D coronary flow between the current iteration (${\bar{Q}}_{\text{in},3D,n_{c}}$) and the previous iteration (${\bar{Q}}_{\text{in},3D,n_{c-1}}$) falls below 1%.

(11) $$E_{Q,n_{c}}=\frac{|{\bar{Q}}_{\text{in},3D,n_{c}}-{\bar{Q}}_{\text{in},3D,n_{c-1}}|}{{\bar{Q}}_{\text{in},3D,n_{c-1}}}$$

Finally, a limit for the maximal number of coupling iterations was set to 20, to avoid possible diverging simulations from iterating indefinitely if no convergence is achieved on either flow or pressure loss. If the maximum number of iterations is exceeded, the simulations are terminated.

### 2.4 Simulations and Analyses

The original 1D model, which includes a lumped stenosis element for computation of stenotic pressure drops, was first studied for three different diameter reductions, 50%, 70% and 80%. Next, for all combinations of varying diameter severities and three lesion types (see Section 2.2.3), all coupling methods were applied. In total, this resulted in 27 1D–3D simulations. The coupling methodologies as described in this chapter all started from the 1D simulations in which the stenosis model of Heinen et al. [9] was used to get an initial estimate for the stenotic pressure loss. The simulation then transitioned to 3D computations as part of the 1D–3D coupling framework.

To evaluate the influence of these initial conditions—referred to as “diseased”—on the final results, an additional set of simulations was performed. In these, the coupling was initiated from a 1D model with a stenosis of 0% diameter reduction (“healthy”), after which a lumped resistance element based on the 3D CFD results was introduced. This “healthy” initialization was only studied for the more complex irregular domains with all coupling approaches, resulting in nine additional simulations.

Results from the three different 1D–3D coupling methods were obtained for comparison. From the 3D simulations, pressure and flow waveforms were extracted at the inlet of the domain, along with the resulting pressure drop across the stenosis ($\Delta p_{\text{sten},3D}$). In the 1D model, pressure waveforms were obtained directly upstream and downstream of the lesion to compute the pressure drop across the stenosis. Flow waveforms were also extracted directly upstream from the stenosis element. Additionally, a distal pressure (location indicated by the black arrow in Figure) was obtained to compute the FFR within the 1D model. The primary outcomes of interest in comparing the different 1D–3D approaches are pressure loss and FFR as a result of the newly defined stenosis model. Secondary objectives were to compare the pressure drop across the stenotic lesion against the original 1D model with the lumped 0D stenosis element. Other outcomes focused on computational metrics such as run time and number of simulated cardiac cycles, to obtain a quantitative metric for the efficiency of each method.

### 2.5 Execution and Infrastructure

The simulations and analyses for the 1D–3D coupling methods of the model were executed on the Ares High‐Performance Computing (HPC) cluster at Academic Computer Center Cyfronet in Kraków. The computational workflow was managed using the Model Execution Environment (MEE) [28], which orchestrated the batch execution of simulation tasks and subsequent postprocessing steps. Each simulation was executed using ANSYS 2024R1, utilizing 24 CPU cores per simulation on nodes equipped with 48‐core, dual Intel Xeon Platinum 8268 2.90GHz CPUs. Memory requirements per simulation were moderate, typically not exceeding 8–10 GB of RAM. Despite the moderate memory footprint, the computational efficiency reported by SLURM consistently reached above 90% for each coupling method, ensuring effective resource utilization. To manage batches of simulation runs across different coupling configurations characterized by coupling mode, lesion type, lesion severity, initialization type, and optionally relaxation factor, we employed SLURM's array job mechanism. MEE facilitated the systematic preparation of input files, submission of array jobs, monitoring of simulation progress, and automated postprocessing of the results.

## 3 Results

### 3.1 Global Hemodynamics 1D Model—Healthy Conditions

The section presents the results for the 1D pulse wave propagation model under healthy conditions, more specifically, a coronary tree without stenosis.

**FIGURE 7:**
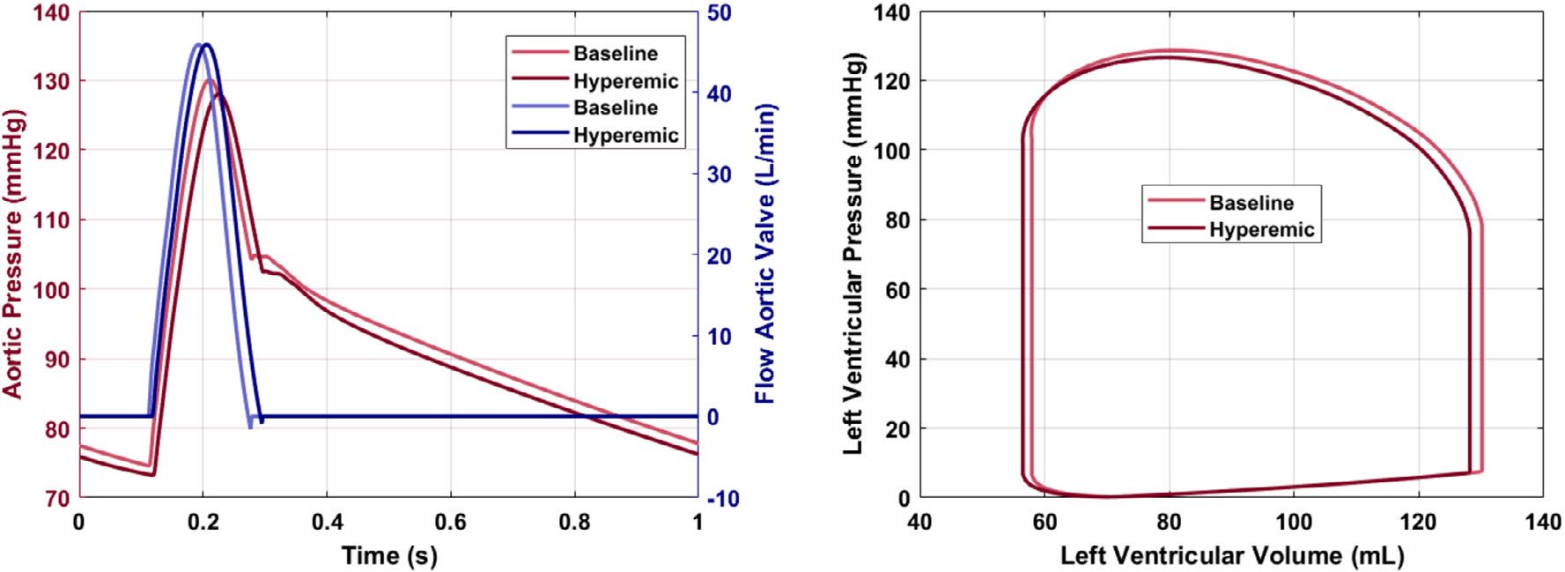
Comparison of aortic and cardiac hemodynamics under conditions of hyperemia and baseline states. The left figure shows the aortic pressure and flow waveforms, and the right figure shows the pressure‐volume loop.

The global hemodynamics of the 1D–0D model were assessed under both resting (baseline) and hyperemic conditions. Figure 7 shows the baseline and hyperemic pressure (in red) and flow (in blue) in the left panel. Both at baseline and during hyperemia, the aortic systolic and diastolic pressures are 129/74 mmHg. The pressure‐volume loops in the right panel of Figure 7 are similar in size, indicating a constant cardiac function, although the volume loop for the hyperemic simulation has shifted slightly to the left. The cardiac output, calculated as the product of stroke volume and heart rate, is 4.3 L/min in the baseline state and increases to 4.7 L/min during hyperemia. In both conditions, the stroke volume, represented by the width of the pressure‐volume loop, remains constant at 72 mL. All hemodynamics are within established physiological limits [29].

**FIGURE 8:**
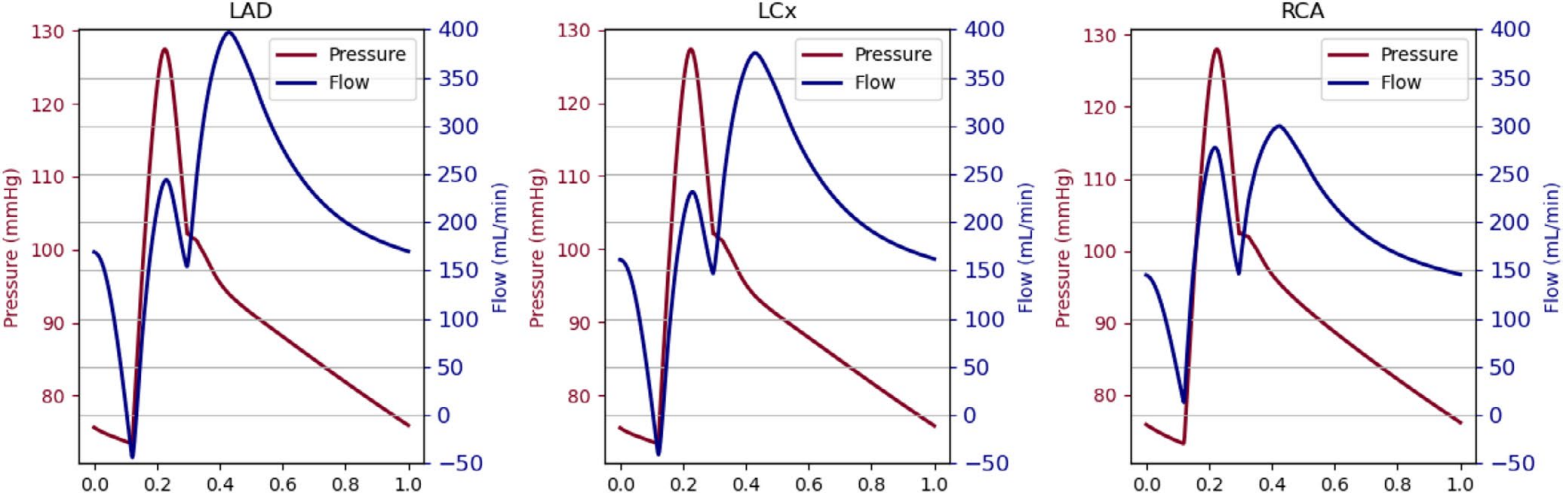
Proximal coronary hemodynamics during hyperemia for each main coronary artery.

Figure 8 illustrates the pressure and flows in the proximal segment of the three epicardial arteries: LAD, LCx, and RCA. The systolic and diastolic coronary pressure in each vessel is equal to 128/73 mmHg, which is consistent with the values reported in [29] and close to the aortic pressure in Figure 7.

The simulated mean hyperaemic flows per epicardial artery are summarized in Table 1. The hyperemic flow values of around 200 mL/min fall within the broad physiological range observed clinically (e.g., 293±102mL/min in the LAD as reported by Fournier et al. [30]). Additionally, the flow waveforms clearly exhibit the diastolic‐dominant flow pattern that is characteristic of coronary circulation, driven by the cyclical compression and relaxation of the myocardium. The coronary flows per epicardial vessel are similar in range, with the highest flow in the LAD and the lowest in the RCA.

### 3.2 Global Hemodynamics 1D Model—Diseased Conditions

**FIGURE 9:**
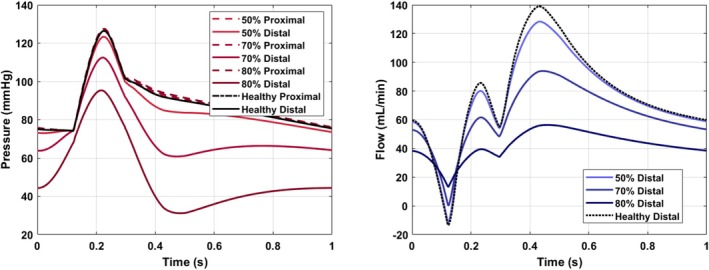
Hemodynamic analysis of the LAD: Pressure (left) and flow (right) waveforms proximal and distal to stenosis during hyperemia.

Diseased conditions in the 1D model were simulated by incorporating the lumped stenosis model from Heinen et al. [9], with varying diameter reductions of 50%, 70%, and 80%. The results of the 1D base pulse wave propagation model are presented in Figure 9. Pressure waveforms were derived at the proximal and distal sites of the stenosis. These waveforms overlap under healthy conditions, while the difference between the proximal and distal waveforms increases for more severe lesions. In contrast, the flow waveforms distal to the stenosis, shown in the right panel of Figure 9, demonstrate a decrease in flow for more severe lesions.

Under healthy conditions, when no stenosis is present at the site later used for lesion placement, the flow equals 77.8 mL/min and the pressure loss over that segment is negligible (~0.0 mmHg), with the FFR close to 1.0. For the three simulated stenoses, the mean pressure drop, flow and FFR were derived and reported in Table 2. Note that stenosis shape is not considered by the standalone 1D model; therefore, identical values are reported below each lesion type. Consistent with the observations in Figure 9, more severe lesions result in lower flow and higher pressure drop. Consequently, the FFR decreases with increasing severity of the lesions, becoming significant (FFR ≤ 0.80) for the lesion with an 80% diameter reduction.

**TABLE 2:** Mean pressure loss, stenotic flow and FFR for each lesion type, severity (DS) and modeling approach.

DS	Coupling method	Symmetric lesion			Eccentric lesion			Irregular lesion		
		$\Delta P_{s}$ (mmHg)	$Q_{s}$ (mL/min)	FFR (−)	$\Delta P_{s}$ (mmHg)	$Q_{s}$ (mL/min)	FFR (−)	$\Delta P_{s}$ (mmHg)	$Q_{s}$ (mL/min)	FFR (−)
50%	Standalone 1D	3.2	75.0	0.95	3.2	75.0	0.95	3.2	75.0	0.95
	Steady coupling	2.0	76.4	0.95	2.4	76.0	0.95	3.6	74.9	0.94
	Cycle‐based coupling	2.2	76.2	0.95	2.4	76.0	0.95	5.2	73.3	0.91
	Time‐step‐based coupling	2.2	76.2	0.95	2.3	76.1	0.95	4.0	74.5	0.93
70%	Standalone 1D	16.6	62.9	0.81	16.6	62.9	0.81	16.6	62.9	0.81
	Steady coupling	13.8	65.7	0.83	15.4	64.3	0.81	24.3	56.2	0.71
	Cycle‐based coupling	14.8	64.9	0.82	16.0	63.7	0.80	25.1	55.5	0.70
	Time‐step‐based coupling	14.7	64.8	0.82	16.1	63.6	0.80	25.0	55.4	0.70
80%	Standalone 1D	39.0	42.6	0.56	39.0	42.6	0.56	39.0	42.6	0.56
	Steady coupling	36.1	45.5	0.58	35.6	45.9	0.59	47.5	35.1	0.46
	Cycle‐based coupling	36.7	45.0	0.58	36.2	45.4	0.58	47.9	34.8	0.46
	Time step‐based coupling	36.7	44.9	0.58	36.2	45.3	0.58	47.9	34.6	0.46

### 3.3 1D–3D Coupling

#### 3.3.1 Global Hemodynamics

**FIGURE 10:**
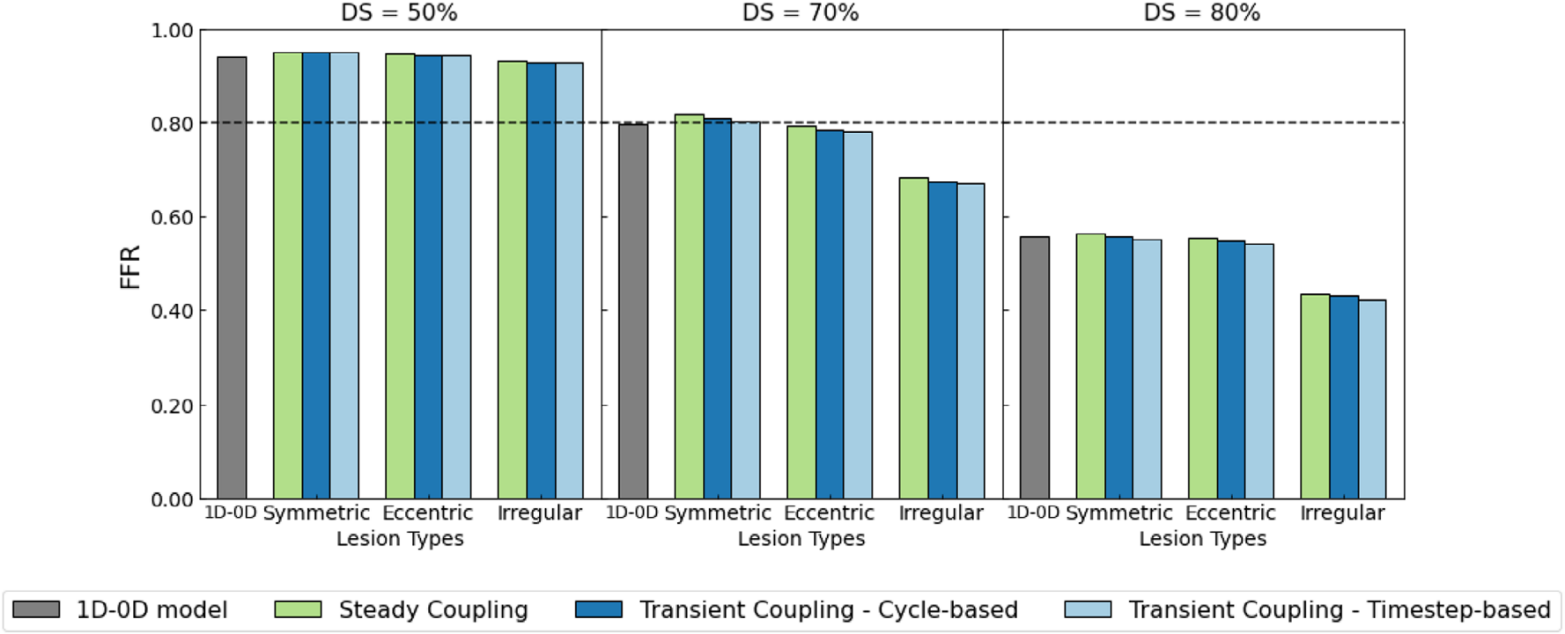
Grouped bar chart of the FFR per method (0D–1D model, steady coupling, transient cycle‐based and transient time step‐based), lesion type (symmetric, eccentric, irregular) and diameter severity (DS = 50%, 70%, 80%).

The three coupling approaches were applied to all combinations of types and severities of lesion. The FFR value was computed per coupling scenario and compared with the conventional 1D–0D model. These results are shown in Figure 10. In addition, pressure loss and stenotic coronary flow were derived from all 1D–3D simulations. These values, together with the exact FFR, are reported in Table 2.

There is minimal variation in FFR between the coupling methods. It was found that the maximum difference in $\Delta P_{s}$ was 1.6 mmHg, in $Q_{s}$ was 1.6 mL/min, and in FFR was 0.03 [−]. Moreover, the original model (1D–0D) results in FFR values similar to those of the 1D–3D methods for symmetric and eccentric lesions—with absolute differences generally below 0.03 (specifically, 0.012–0.029). For irregular lesions, the lumped stenosis element tends to overestimate FFR compared to multiscale simulations. At 50% severity, differences are modest (0.004–0.025), but increase to maximally 0.10 at 70%–80% severity. In accordance with the lower FFR for irregular lesions, Table 2 demonstrates a higher pressure loss and lower flow for these lesions.

#### 3.3.2 Convergence Time‐Step‐Based Coupling

**FIGURE 11:**
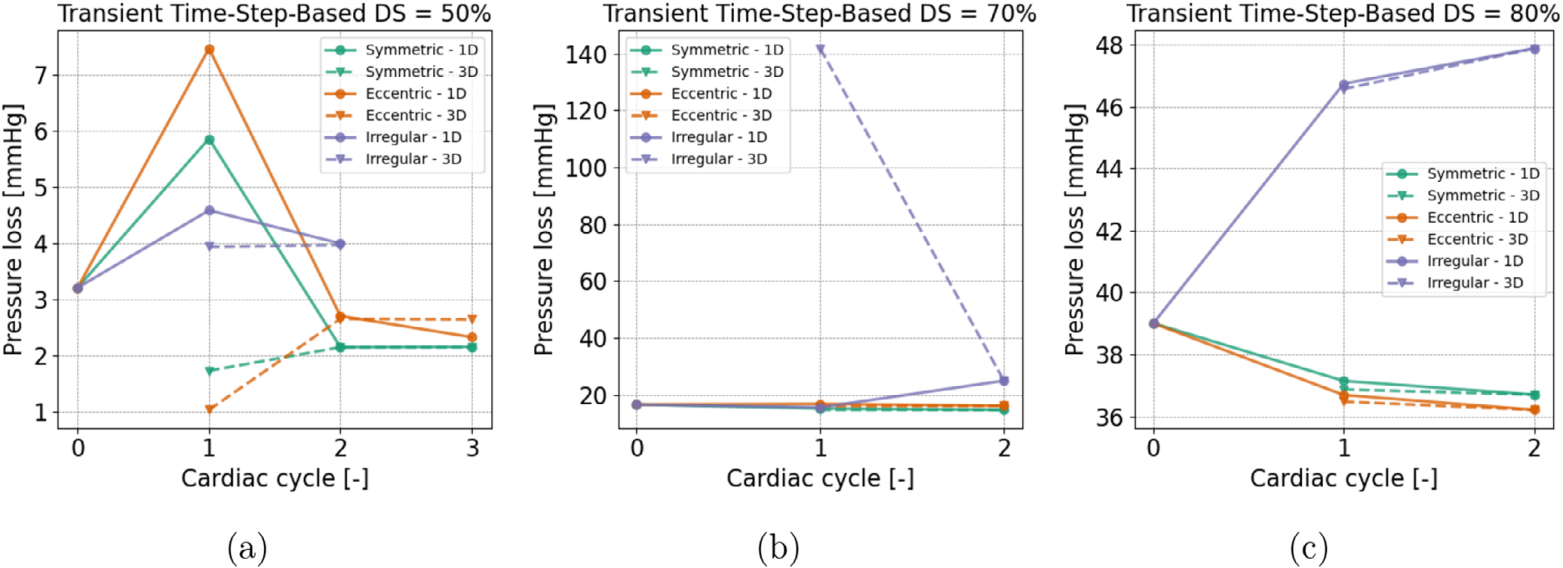
Time step‐based coupling: Number of cycles to convergence. Pressure loss across the lesion is shown over the cycles for different lesion geometries (symmetric, eccentric, irregular) and severities (DS = 50%, 70% and 80%). The markers indicate pressure loss in both the 1D and 3D domains as updated throughout the coupling process.

**FIGURE 12:**
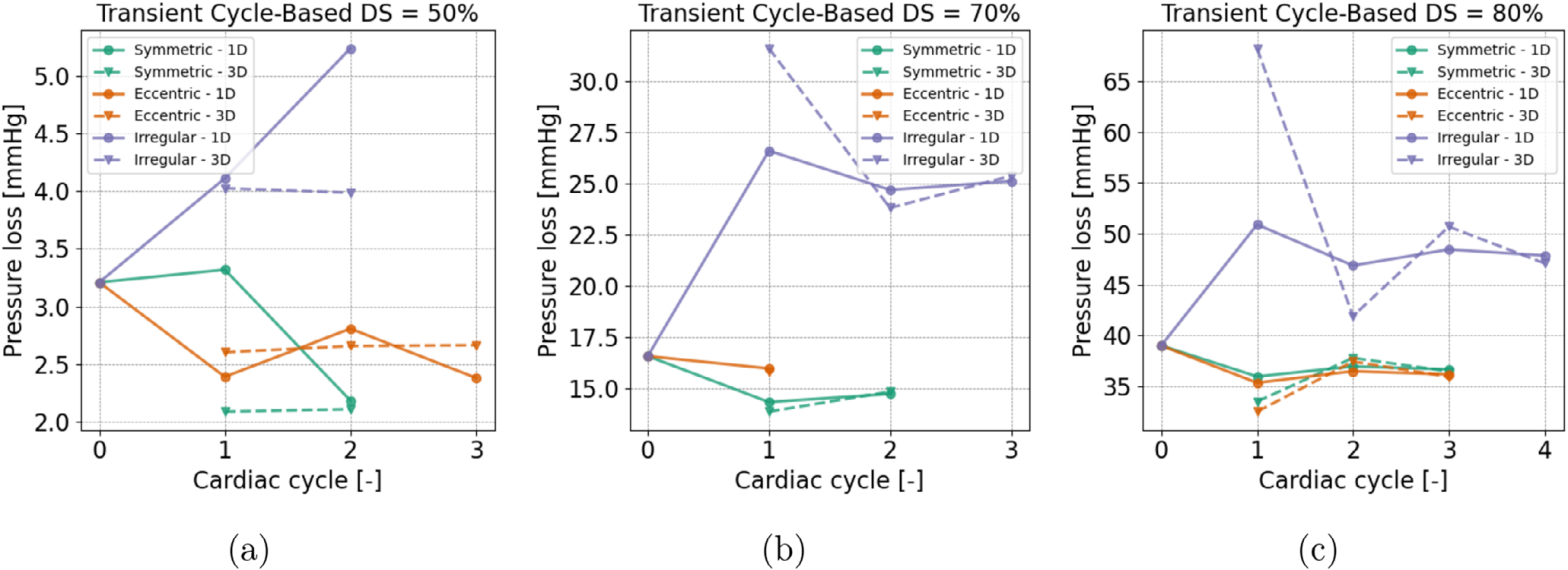
Cycle‐based coupling: Number of cycles to convergence. Pressure loss across the lesion is shown over the cycles for different lesion geometries (symmetric, eccentric, irregular) and severities (DS = 50%, 70% and 80%). The markers indicate pressure loss in both the 1D and 3D domains as updated throughout the coupling process.

**FIGURE 13:**
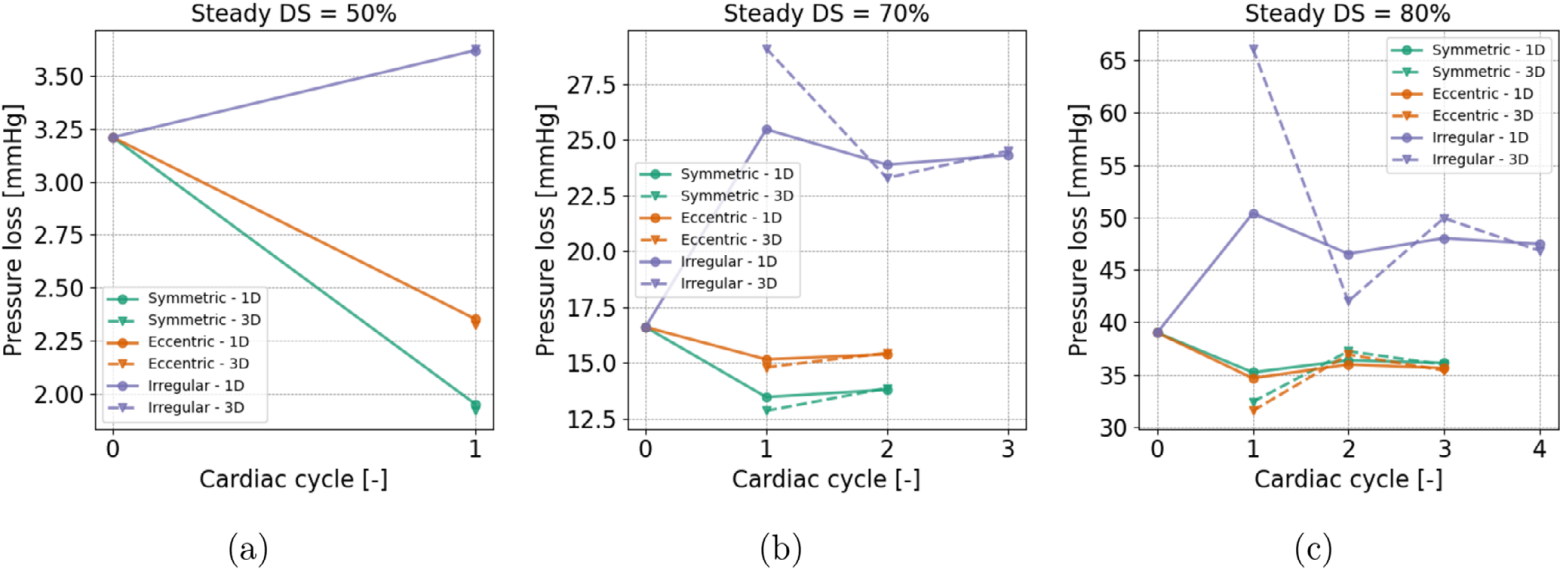
Steady coupling: Number of cycles to convergence. Pressure loss across the lesion is shown over the cycles for different lesion geometries (symmetric, eccentric, irregular) and severities (DS = 50%, 70% and 80%). The markers indicate pressure loss in both the 1D and 3D domains as updated throughout the coupling process.

The time‐averaged pressure losses for the 1D and 3D models were calculated for each coupling cycle. Figure 11 shows the mean pressure loss per coupling cycle for the time step‐based coupling. Cycle 0 here responds to the results obtained right before the onset of the 1D–3D coupling scheme (this also holds for Figures 12 and 13). Convergence is reached rapidly for all geometry types, ranging from two to three cycles. The Eccentric 50% lesion geometry simulation, however, shows that after the second cycle, the difference between the 1D and 3D pressure increases slightly.

#### 3.3.3 Convergence Cycle‐Based Coupling

For the cycle‐based coupling methods, the results in each coupling cycle are shown in Figure 12. For the lesions with a high severity (70% and 80%), the results converged between 1 and 4 cycles, with the irregular lesions taking the most cycles to complete. For the 50% lesion, the simulations converged faster, however, there is a larger mismatch between the 1D and 3D pressure drop. By closely examining the results for the irregular 50% lesion depicted in Figure 12, it can be observed that during cardiac cycle 1, the pressure loss values between the 3D and 1D models are already quite similar, with a difference around 0.1 mmHg. At these lower pressure loss levels, the error in the pressure drop is approximately 2%–3%, which exceeds the 2% cutoff defined in Section 2.3.4. Consequently, this necessitates the coupling approach to run the model for an additional cycle based on criterion [10], after which it converges according to the flow criterion [11]. Similar behavior is observed for the eccentric 50% lesion.

#### 3.3.4 Convergence Steady Coupling

The time‐averaged pressure losses for the steady coupling method are shown in Figure 13. For the symmetric and eccentric lesions, a fast convergence is observed, that is after two to three coupling cycles the 1D pressure loss matches the 3D pressure loss, similarly does the flow. For a lesion of 50%, all coupling methods converged within one cycle, meaning only one 3D simulation was needed. For the irregular lesions, the approach generally takes longer to converge, up to four coupling cycles. For symmetric and eccentric lesions, it can be seen that the pressure loss becomes slightly lower compared to the initial value in cycle 0, whereas for irregular stenoses the pressure loss increases.

### 3.4 Computational Time and Efficiency

**FIGURE 14:**
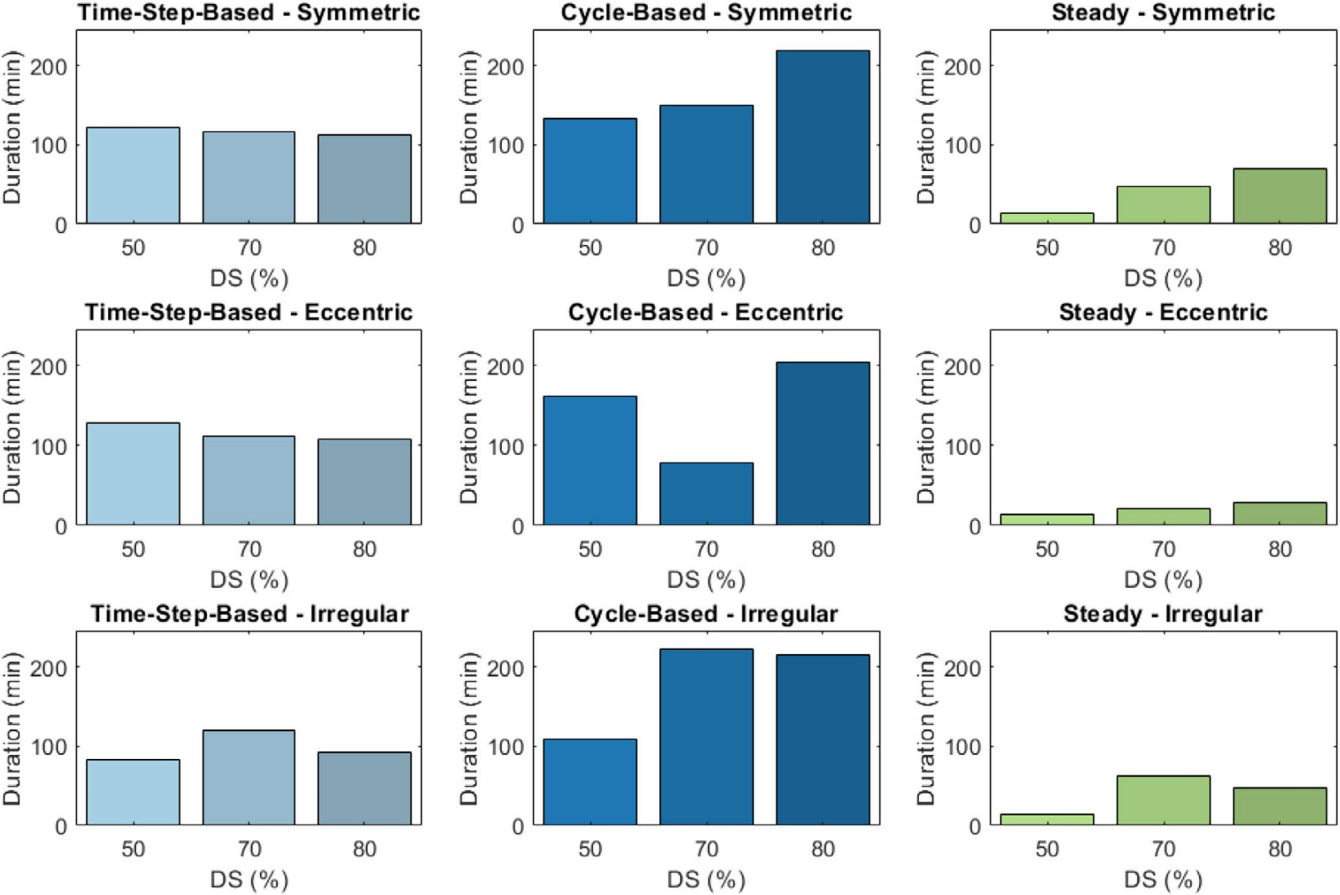
Overall duration of all simulations in minutes.

The computational times for different coupling strategies were evaluated. Steady‐coupled simulations were the fastest, with durations ranging from approximately 10 min to 1 h. In contrast, cycle‐based simulations were the most time‐consuming, taking about one to three and a half hours. Time‐step‐based simulations fell in between, typically running from 1 to 2 h. Figure 14 provides an overview of these simulation times.

### 3.5 Initialization

**FIGURE 15:**
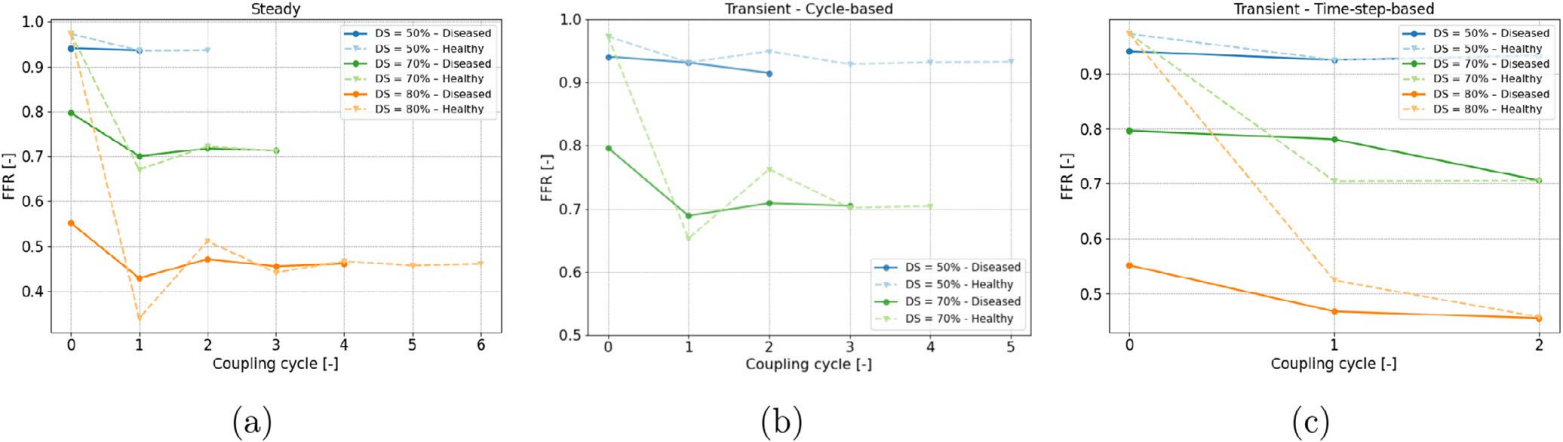
Effect of initial lumped stenosis severity on convergence for simulations with a diffuse lesion.

The 1D–3D coupling results displayed so far were obtained using the 1D–3D coupling methods, in which the 1D model was run with the original stenosis element before transitioning to the 3D model. Alternatively, multi‐scale modeling can begin with the 1D model in a healthy state (by setting the lesion severity to 0%), without any assumptions about expected stenotic behavior. Figure 15 illustrates the comparison between initialising the 1D–3D coupling from a healthy 1D–0D model and a diseased model.

For cycle‐based and steady simulations, initializing with a diseased 1D model results in faster convergence, typically requiring one or two fewer cycles. In contrast, the timestep‐based approach is unaffected by the type of initialization; all simulations converge within two cycles, regardless of whether the initialization is from a healthy or diseased model.

The final FFR values after convergence of the coupling schemes are reported in Table 3. By comparing the converged FFR between the diseased and healthy initialization for each lesion, most values are equal and for some differences are below 0.02. Only for the 80% lesion, the cycle‐based coupling with “healthy” initialization became unstable; therefore, these results were excluded from the analysis.

**TABLE 3:** Final FFR values for diffuse lesions with two different initialization types: Healthy and diseased. The severity of the lesions was 50%, 70%, and 80%.

Initialization	Steady			Cycle‐based			Time step‐based		
	50%	70%	80%	50%	70%	80%	50%	70%	80%
Diseased	0.94	0.71	0.46	0.91	0.70	0.46	0.93	0.71	0.45
Healthy	0.94	0.71	0.46	0.93	0.70	0.57	0.93	0.71	0.45

## 4 Discussion

In this work, we explored various strategies for integrating a three‐dimensional CFD model into a combined 1D–0D wave propagation model of the coronary tree. These methods were designed to enhance the accuracy of lesion‐specific pressure drop predictions while retaining computational efficiency. Thereto, we compared the performance of these approaches both against one another and with a faster, albeit less precise, 1D–0D model. Furthermore, we investigated the necessary amount of simulated cardiac cycles until convergence, the overall stability, and the robustness of each method.

A 1D–0D pulse wave propagation model was chosen for its ability to efficiently simulate pressure and flow waveforms throughout the vasculature, both upstream and downstream of cardiovascular lesions. This makes it a physiologically consistent and computationally efficient boundary condition for multi‐scale modeling. Due to its computational speed, it could also be used in model‐based decision support, making it well‐suited for potential clinical applications. While the model reproduces realistic coronary waveforms under normal circumstances (Figures 7 and 8), it is crucial to assess its applicability to the heterogeneous population with coronary artery disease. Initially, the model included a simple lumped stenosis model, derived from an idealistic symmetric lesion, to estimate coronary pressure losses. Consequently, it was replaced by a 3D model with various stenosis geometries. Results showed, as expected, that different stenosis geometries produce varying pressure losses (Figure 10). For the simpler (symmetric and eccentric) lesions, the 1D–0D approach proves equally accurate compared to more complex 1D–3D simulations (Table 2). However, the discrepancy in FFR values for severe diffuse lesions reached approximately 0.10 [−], which is clinically significant [31]. This highlights the limitations of standalone 1D models in capturing complex lesion behavior and underscores the value of 1D–3D coupled models, which combine computational efficiency with improved prediction accuracy across a range of lesion types.

### 4.1 Comparison of the Coupling Methodologies

The three coupling methods introduced in this work produced nearly identical values for the time‐averaged pressure drop, time‐averaged flow, and FFR. The maximum differences in FFR were 0.03, which is roughly equivalent to the repeatability error of 0.02 for invasively measured FFR [31]. From the results depicting the necessary amount of cardiac cycles until convergence, we found that the steady coupled simulations were overall the fastest, with times increasing from 10 min to 1 h as the diameter severity increased. In contrast, the time‐step‐based and cycle‐based coupling methods required significantly more time, averaging at 1 to 2 h, even when using 34 CPUs. Additionally, both the cycle‐ and time‐step‐based coupling methods were less robust than the steady coupling methods, as they showed divergence over time for some cases. Initialization by the lumped stenosis model had positive effects on computational efficiency.

**FIGURE C1:**
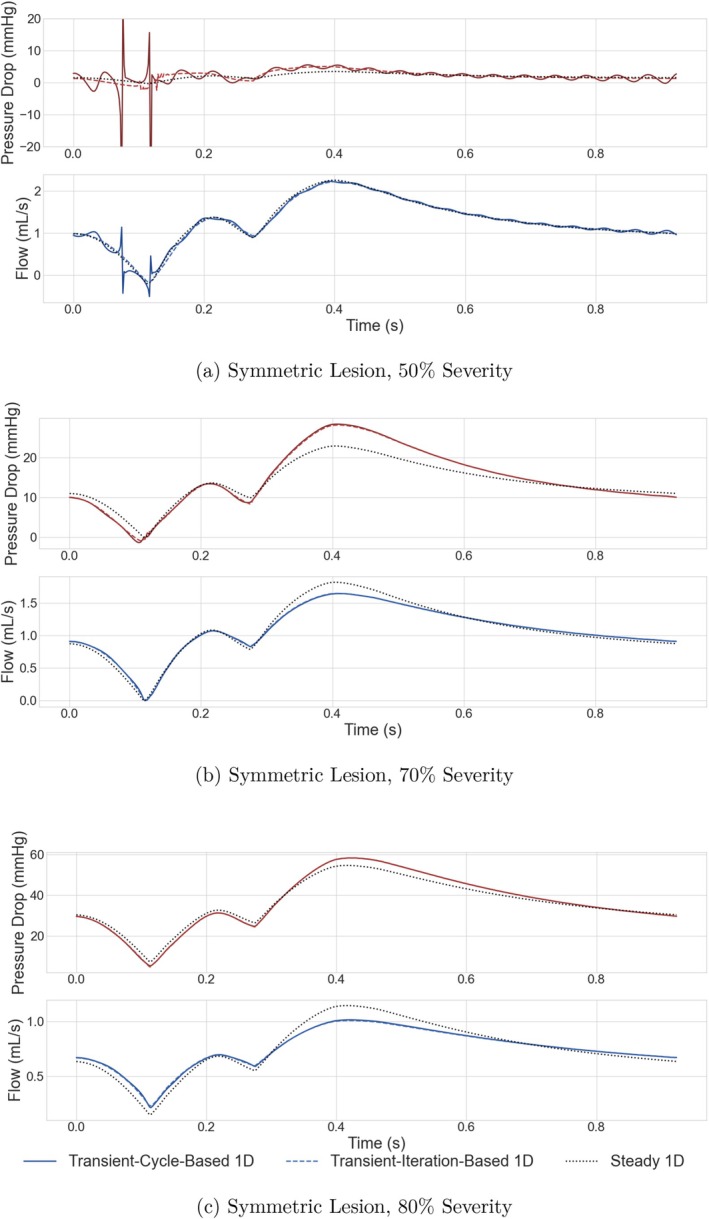
Pressure drop and flow for symmetric lesions at 50%, 70%, and 80% severity (1D simulations).

Notably, the FFR derived from the 1D–3D coupled approaches was consistent across all three methods, as illustrated in Figure 10. When coupled with a steady 3D simulation, the flow and pressure waveforms in the 1D model showed slight deviations from those produced when coupled to transient 3D simulations (see Appendix C). As a typical example, Figure C1b shows that the transient 1D pressure drop deviated by up to ~24% in peak values and ~7% in average values compared to the steady solution. For flow, peak differences remained below 10%, with average deviations under 1.5%. Although differences between the steady and transient cases are evident in the 1D model, Figure 10 demonstrates that the mean flow and FFR remain consistent across these simulations. This consistency suggests that for rapid FFR prediction using a 1D–3D coupled methodology, a steady simulation is sufficient, as our results indicate that transient effects can be neglected when considering a time‐averaged FFR. However, suppose the focus were on coronary flows or other physiological values at specific time instances or periods (e.g., diastole vs. systole). In that case, the results will be impacted by neglecting transient effects. The velocity field responds directly to time‐dependent changes such as fluctuations, vortices, and flow separation, which can lead to more significant variations between the simulation types [32]. In contrast, pressure fields tend to be smoother and more spatially uniform, acting as a driving force rather than a responsive variable. As a result, transient simulations are better at capturing dynamic phenomena that impact flow and might be necessary if interests lie beyond the time‐averaged FFR.

### 4.2 Instabilities During Coupling

Instabilities were observed when examining the flow and pressure waveforms more closely, as shown in Appendix C. It was seen that both transient coupling methodologies do give rise to some oscillations or instabilities. The instabilities that arise during cycle‐based and time‐step‐based coupling schemes can be attributed to the flow becoming negative (backflow). The coronary arteries exhibit a diastolic dominant flow profile due to the contraction of the heart muscle [29]. To model this external myocardial contractile pressure in the 1D model, a pressure was applied to the external compliance nodes of the coronary windkessel elements. In cases with stenotic lesions of DS=50%, the flow can become negative during peak systole. This negative flow translates to a negative resistance value at those time steps, leading to spikes in the pressure and flow signals. At the same time, before the change in flow direction from positive to negative, the flow approaches zero, possibly resulting in infinite resistance values. As a result, this causes disruptions in the computed pressure and flow at that specific moment, resulting in spikes in both pressure and flow waveforms. Consequently, for cycle‐based coupling, these spikes complicate the Fourier fitting process, leading to significant oscillations in the flow and pressure waveforms for the ideal 50% lesion.

These instabilities were not observed for other stenosis severities or under physiological flow conditions where the flow remained positive throughout the cardiac cycle. Therefore, they do not affect the overall findings of the study. On the contrary, their occurrence highlights a limitation of our current transient coupling schemes under flow‐reversal conditions, supporting the conclusion that the steady‐state coupling method is the most robust and computationally efficient approach among those investigated. Although initialization with the lumped stenosis element could already be the solution to this problem. This observation further reinforces the comparative nature of this work, where identifying both the strengths and limitations of each coupling strategy is essential to assessing their practical applicability.

To enhance stability between the multi‐domain models, it is recommended to improve the 1D model by incorporating an iterative scheme, such as the Newton–Raphson method, particularly for the time‐step‐based approach. In the cycle‐based coupling method, some progress has been made by running the model through several cycles; however, these cycles have not been checked for convergence.

For time‐step‐based coupling, under‐relaxation was already applied to counter the oscillations. However, computing the resistance element as $R=\frac{p}{q}$ still risks infinite values when flow q approaches zero. This can be mitigated by imposing a minimum flow threshold—ensuring q never falls below a physiologically meaningful floor—or by capping resistance at a realistic upper limit and smoothing the resistance update via under‐relaxation, adding a small regularization term to the denominator. Another option could be including a small parallel compliance element downstream, which further guarantees that true zero flow is never reached.

A study by Bertoglio et al. showed that implicit over explicit coupling of a three‐element Windkessel as a CFD outlet improved the stability of the CFD solution [33]. In this paper, the coupling was done explicitly between the 1D and 3D models; hence, implicitly linking both models might result in more robust transient coupling schemes. Furthermore, various other coupling schemes might prove to be more adequate; however, the implementation might be less straightforward and more complex. Using more strongly coupled Dirichlet–Neumann coupling schemes or the GMRES (generalized minimal residual method) could potentially address stability issues sometimes encountered with explicit coupling [34, 35].

### 4.3 Limitations of Study

The current study exhibits certain limitations. First, the various coupling schemes were tested solely with synthetic stenosis models, which raises questions about how accurately these reflect the characteristics of real‐world stenoses. Secondly, hyperemia, the state of maximal microvascular dilation, is a prerequisite for reliable FFR computation. In our current 1D model, we mimic it by dividing every outlet resistance by three—an approach that ignores vessel‐ and patient‐specific autoregulatory adjustments downstream of a stenosis [22]. In moderate lesions, a drop in distal pressure triggers further vasodilation, lowering resistance and partially restoring flow; this, however, increases the translesional pressure drop and therefore reduces FFR. In very severe stenoses, downstream arterioles are already maximally dilated at rest, so no additional reserve remains and hyperemic flow—hence FFR—stays essentially unchanged. A more physiological scheme would make the resistance‐reduction factor itself a function of lesion severity (smaller for critical stenoses, larger for mild ones), but we have not yet implemented these coupled autoregulatory dynamics—so our model may underestimate flow in severe cases. Note that simply increasing the global hyperemia factor would raise the mean hyperemic flow closer to the clinically measured average, as seen in Figure 8 [30]. Third, our model assumes preserved global cardiac function, which may not hold in cases of severe coronary artery disease where ischemia can impair left ventricular contractility and alter the pressure‐volume loop. Furthermore, while coupling a 3D CFD model with a 1D framework yields detailed pressure and flow distributions, it increases simulation times to over 10 min. This diminishes one of the core advantages of the 1D–0D approach—its computational speed—which is critical for seamless integration into clinical workflows where time and efficiency are paramount. Moreover, a parabolic inlet velocity profile was assumed for the 3D simulations, justified by the Womersley number of approximately 2. However, in vivo coronary flows are typically flatter and more plug‐like [36]. This was partly accounted for in the 1D model using the approximate velocity profile of Bessems et al. [37] (see also our previous work [20] for a more detailed explanation of its implementation within the 1D pulse wave propagation model). Nevertheless, this correction was not applied in the 3D model, which may reduce the physiological accuracy of predicted wall shear stress and local flow patterns. Additionally, blood was modeled as an incompressible fluid and the coronary arteries were assumed to have rigid walls. While such assumptions simplify the analysis, they neglect the interaction between pulsatile flow and wall compliance. A previous study by Albadawi et al. reported that in stenotic coronary arteries, the rigid‐wall assumption underestimated wall shear stress values compared with an elastic‐wall model, with differences exceeding 10% at the stenosis throat [38]. This highlights that vessel compliance can substantially influence local hemodynamic parameters in stenotic regions, and should be considered for more physiologically accurate modeling. Incorporating lesion‐specific material properties through FSI techniques allows for a more realistic representation of stiffness variations and their impact on coronary physiology. FSI within a 1D–3D modeling framework has been previously explored by Formaggia et al. [12], Beulen et al. [14], and Blanco et al. [18].

## 5 Conclusion

Three different methods were implemented to couple a 3D CFD model of a stenosis with a 1D model of the entire coronary tree. One approach used steady‐state CFD simulations based on mean flow values from the transient 1D model, while the other two relied on transient 3D simulations with data exchange either at each time step or at the end of a cardiac cycle. All three methods produced similar results, indicating that the steady‐state approach—being significantly faster—is the most efficient and practical option for characterizing stenotic pressure loss using explicit 1D–3D coupling. For irregular lesions, our high‐fidelity 1D–3D coupling simulations yielded pressure loss predictions that significantly diverged from those of the simplified lumped‐stenosis model in the 1D–0D framework, underscoring the added value of localized 3D modeling in complex cases.

## Funding

This work was supported by the European Union's Horizon 2020 Research and Innovation Programme through the Research and Innovation Actions “In Silico World: Lowering Barriers to Ubiquitous Adoption of In Silico Trials”, 101016503. Polish High‐Performance Computing Infrastructure PLGrid (HPC Center: ACK Cyfronet AGH), PLG/2024/017022, PLG/2024/017108.

## Conflicts of Interest

The authors declare no conflicts of interest.

## Figures and Tables

**FIGURE B1:**
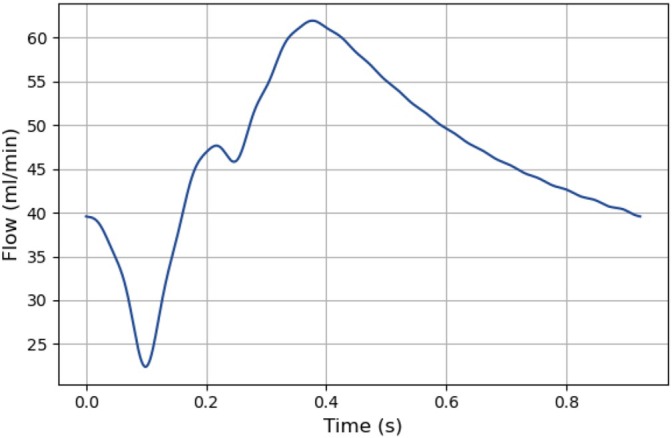
Inlet coronary flow profile for the dependency analysis.

**FIGURE B2:**
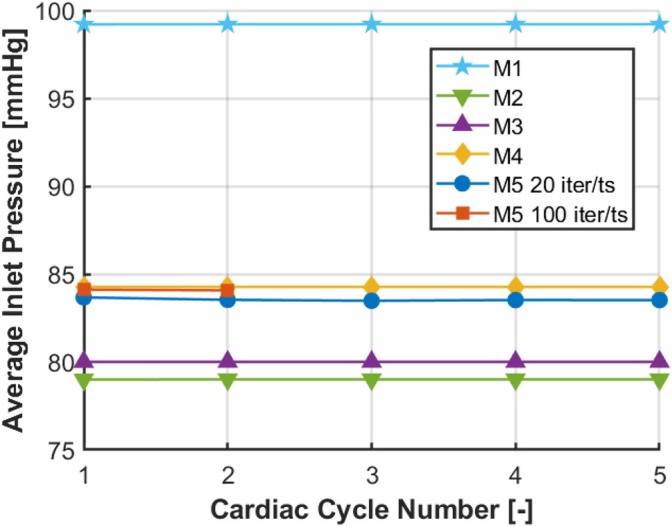
The area‐averaged inlet pressure in each cardiac cycle for each mesh. Please note that for M5, the results of 20 iterations/time step and 100 iterations/time step are depicted here.

**FIGURE B3:**
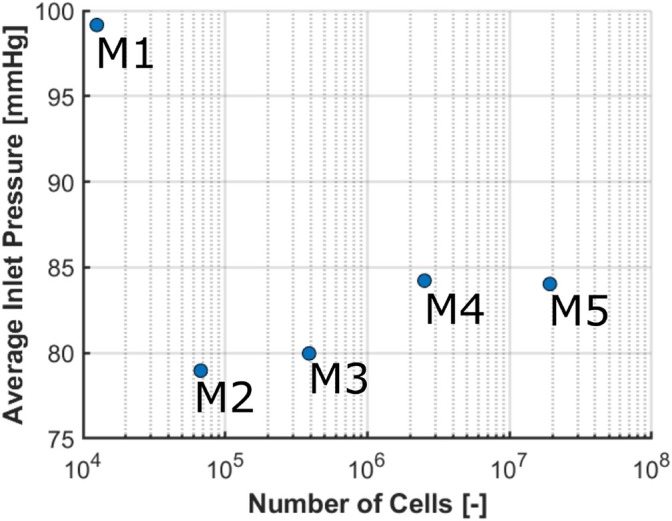
The area‐averaged inlet pressure in the final cardiac cycle for various meshes for different numbers of cells.

**FIGURE B4:**
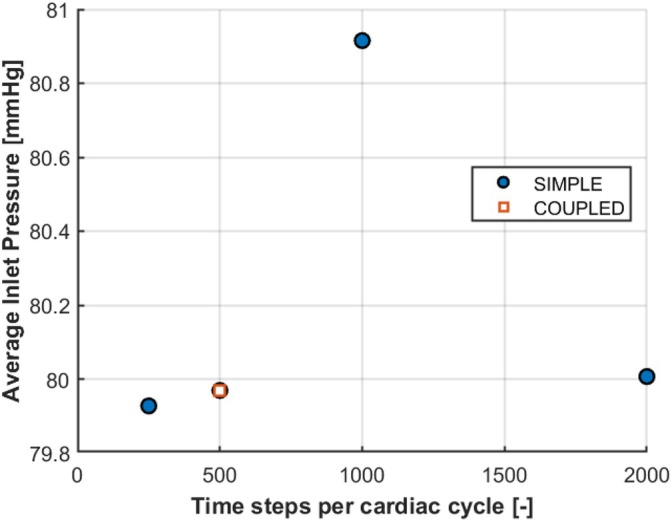
Mean pressure loss in the 2nd cardiac cycle for different time step sizes.

**FIGURE C2:**
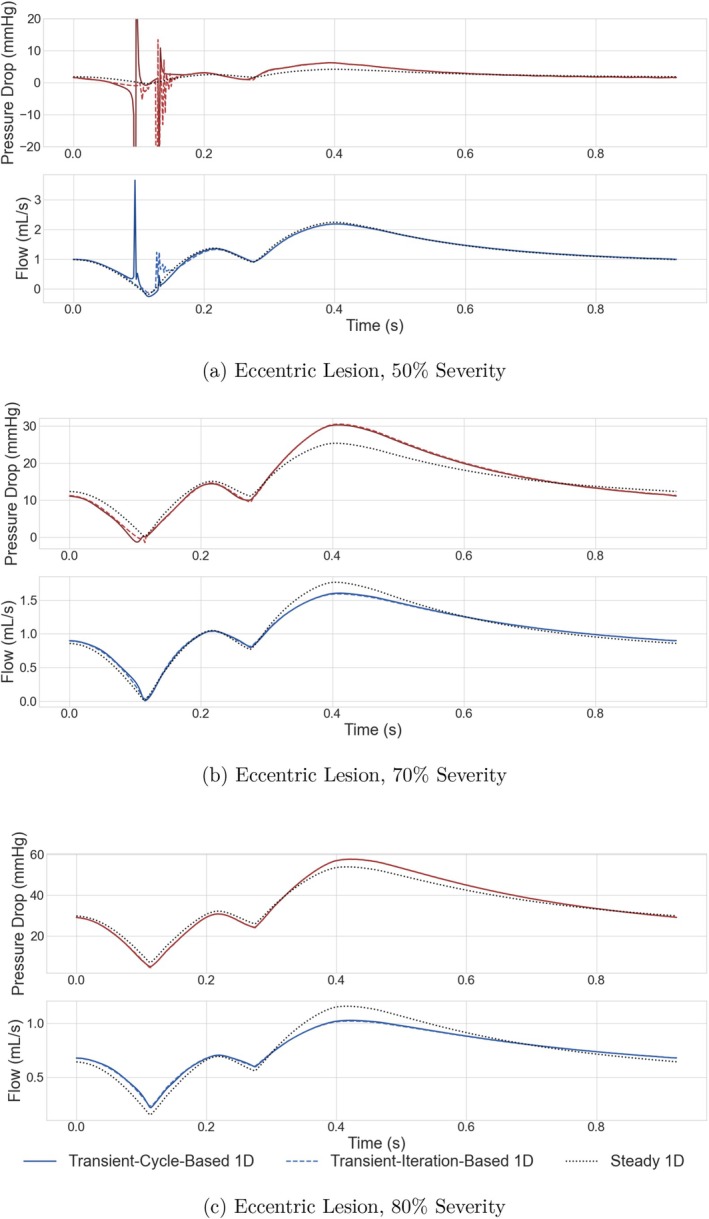
Pressure drop and flow for eccentric lesions at 50%, 70%, and 80% severity (1D simulations).

**FIGURE C3:**
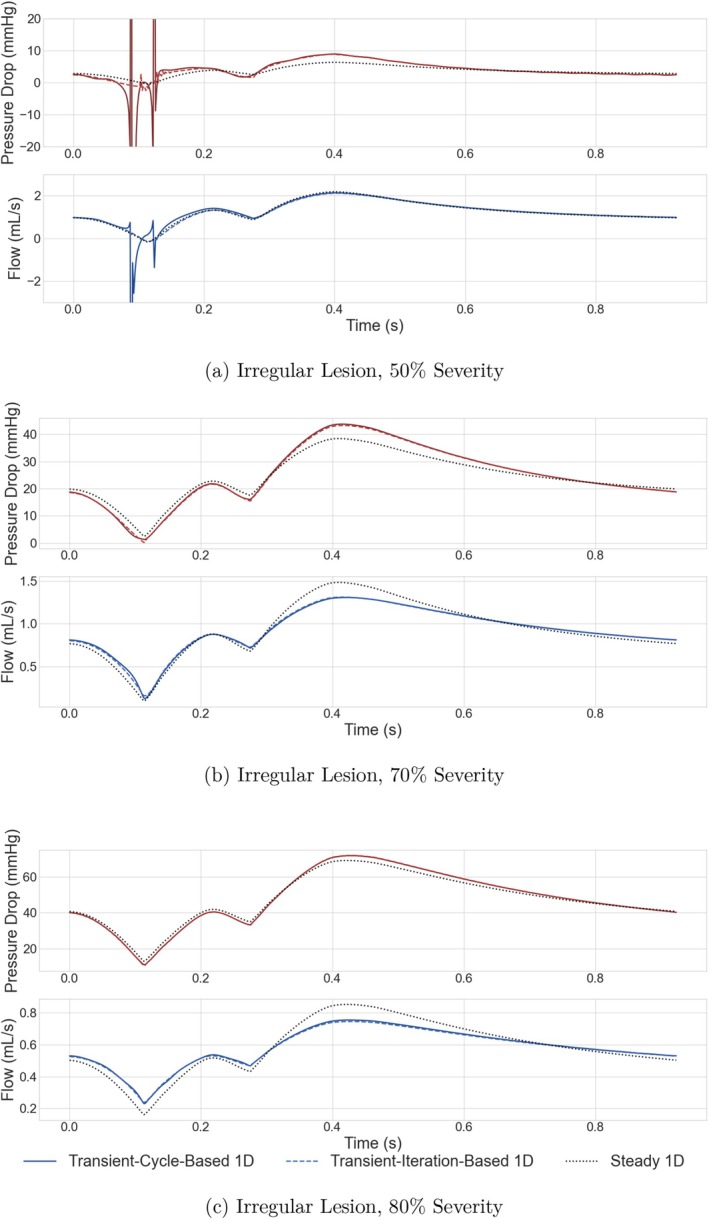
Pressure drop and flow for irregular lesions at 50%, 70%, and 80% severity (1D simulations).

**FIGURE C4:**
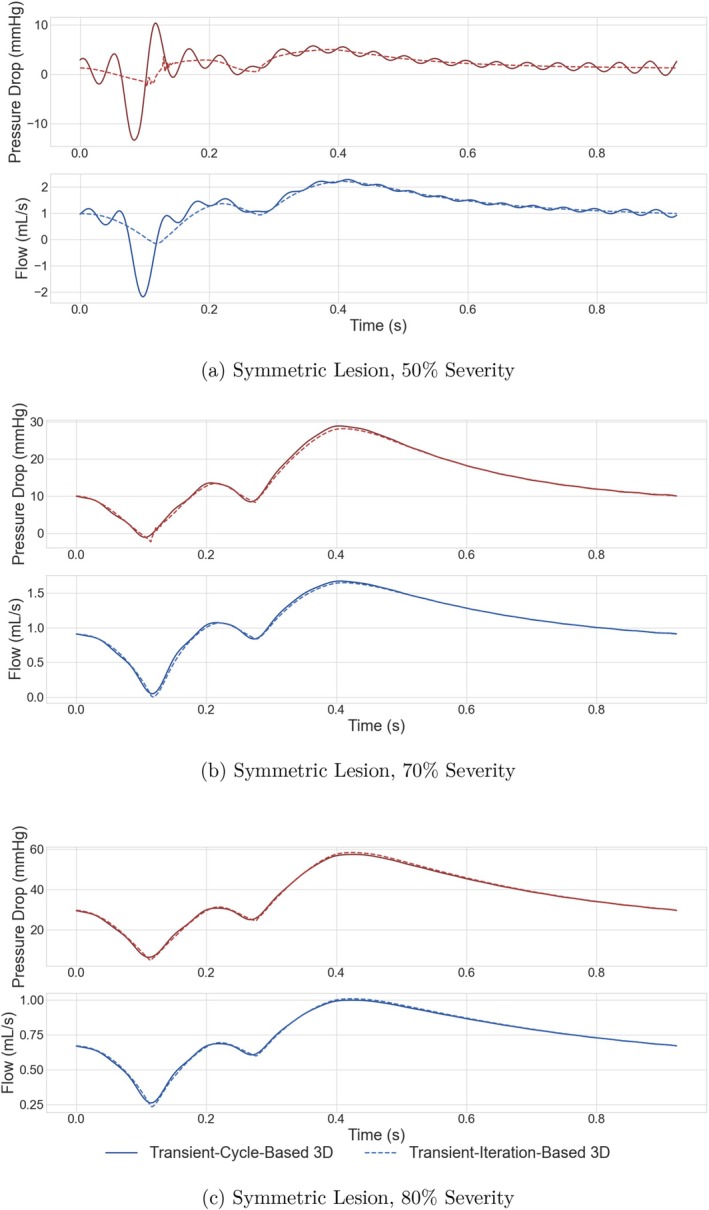
Pressure drop and flow for symmetric lesions at 50%, 70%, and 80% severity (3D simulations).

**FIGURE C5:**
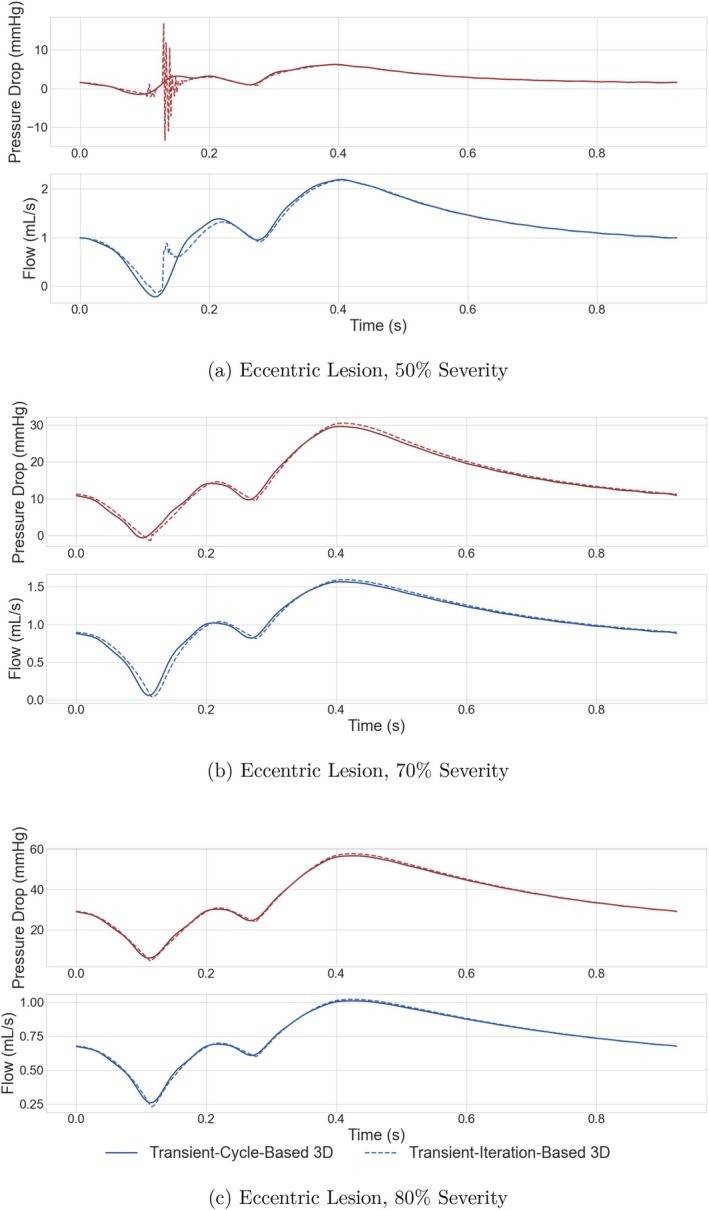
Pressure drop and flow for eccentric lesions at 50%, 70%, and 80% severity (3D simulations).

**FIGURE C6:**
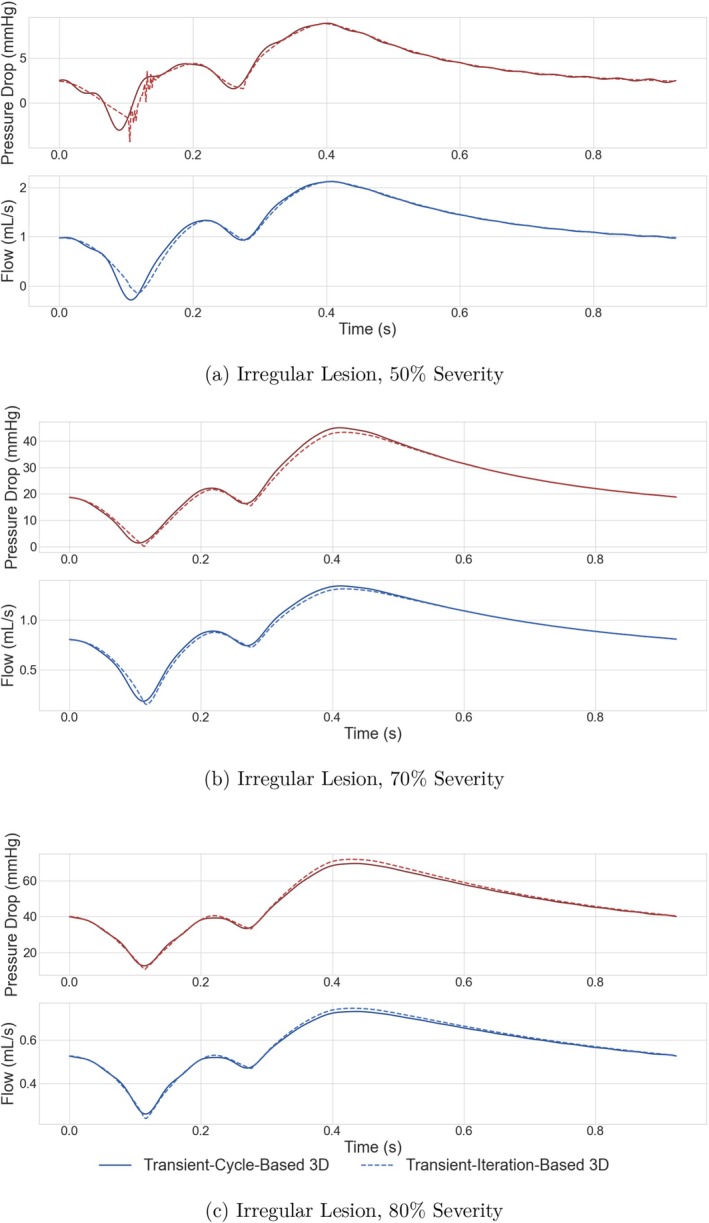
Pressure drop and flow for irregular lesions at 50%, 70%, and 80% severity (3D simulations).

## Data Availability

The data that support the findings of this study are available from the corresponding author upon reasonable request.

## Appendix A Mesh Information—3D Lesion Geometries

Details on the meshes for each geometry were derived from Fluent Meshing and are reported in Table A1. Fluent Meshing provides the number of nodes and faces for both the interior and boundary regions. The numbers shown in Table A1 represent the sum of the interior and boundary counts for both nodes and faces.

**TABLE A1:** Mesh information for the volumetric domains derived from the nine different stenosis geometries. The total cell count, face count, and node count are reported.

Lesion type	DS (%)	# Cells	# Faces	# Nodes
Symmetric	50	445,558	3,004,841	2,492,141
Symmetric	70	458,390	3,095,596	2,563,023
Symmetric	80	417,054	2,809,972	2,333,359
Eccentric	50	410,048	2,757,724	2,294,545
Eccentric	70	405,824	2,729,589	2,271,170
Eccentric	80	394,593	2,653,177	2,208,811
Irregular	50	387,188	2,601,503	2,166,481
Irregular	70	383,071	2,573,123	2,143,571
Irregular	80	387,815	2,606,892	2,170,299

## Appendix B Mesh, Time Step, and Cycle Convergence

The dependence of results from the 3D model on time step size, mesh dimensions and cardiac cycles was analyzed for the most severe lesion, that is, irregular lesion with a diameter reduction of 80%. A coronary inflow waveform, derived from the 1D model with a predefined 80% stenosis at the specified location, was used as the inlet boundary condition. The inlet flow profile is depicted in Figure B1, showing a mean flow rate of approximately 48 mL/min.

Five mesh configurations were tested, by decreasing the minimal and maximal cell length in steps of two. The smallest cell length was set to $2\times 10^{-5}$ m, whilst the largest was $32\times 10^{-5}$ m. Table B1 details the number of cells, faces, and nodes for each mesh configuration. Simulations were run with 500 timesteps per cardiac cycle and a time step size of 1.84 ms, using settings consistent with those described in Section 2.2.3. For models M1–M4, simulations were performed over five cardiac cycles to evaluate the influence of initialization on the results. A maximum of 200 iterations per time step was allowed. For computational efficiency, however, the finest mesh M5 was tested in two configurations: five cycles with 20 iterations per time step and two cycles with 100 iterations per time step.

Figure B2 illustrates the mean pressure ($\Delta \bar{P}$) loss per cardiac cycle for various mesh sizes. For meshes M1–M4, there is no discernible difference between cycles. However, for mesh M5, a 0.1 mmHg difference is observed when using 20 iterations per time step, with the difference at the fifth cardiac cycle being only 0.01 mmHg. When using 100 iterations per time step, there is a 0.03 mmHg difference between the first and second cycles. These results indicate minimal difference in pressure loss across cardiac cycles, and that the different number of iterations for M5 has only a small impact on cycle‐to‐cycle variation. Both configurations for M5 produced a relatively similar pressure drop of 83.5 and 84 mmHg. Based on this mesh analysis, we simulate two cardiac cycles with 100 iterations per time step in the 3D model to avoid any cycle‐to‐cycle variation due to initialization effects and to increase accuracy with a higher number of iterations.

**TABLE B1:** Description of the mesh configurations in the mesh dependence analysis, defined by the maximum cell length and the number of cells, faces and nodes.

Mesh	Max cell length (m)	Nr of cells	Nr of faces	Nr of nodes
M1	32 × 10	12,495	80,824	65,520
M2	16 × 10^5^	67,255	445,468	365,643
M3	8 × 10^−5^	388,613	2,615,916	2,147,216
M4	4 × 10^−5^	2,520,442	17,154,572	14,095,224
M5	2 × 10^−5^	19,210,726	132,005,019	109,516,972

Figure B3 shows the mean pressure loss for various meshes in the second cardiac cycle. Variations in mesh size have a more pronounced effect on the computed pressure loss. As the number of cells increases, the pressure drop gradually converges. For the coarsest mesh, M1 (12,495 cells), the relative difference compared to the finest mesh, M5 (19,210,726 cells), is 18.0%, as shown in Table B2. For mesh M2 (67,255 cells), the difference is reduced to 6.0%. Ultimately, mesh M3 (388,613 cells) was chosen because it resulted in only a 4.8% difference. The corresponding pressure drop difference of approximately 4 mmHg between the final mesh refinements is slightly higher than the commonly accepted clinical drift threshold of 3 mmHg, above which re‐equalization is typically recommended [39]. However, since the current focus is on comparing numerical behavior across different 1D–3D coupling strategies rather than accurately predicting FFR, this level of variation is acceptable for now. The chosen mesh offers a good balance between accuracy and computational cost. While mesh M4 (2,520,442 cells) produced a smaller error of just 0.2%, its significantly higher computational cost outweighed the marginal gain in accuracy. In future applications requiring precise FFR prediction, the mesh convergence tolerance should be tightened to stay within the clinically relevant threshold.

**TABLE B2:** Relative difference of average inlet pressure compared to the finest mesh.

Mesh	Nr of cells	Relative difference
M1	12,495	18%
M2	67,255	6.0%
M3	388,613	4.8%
M4	2,520,442	0.2%
M5	19,210,726	0%

The time step analysis was performed using simulations with different numbers of time steps per cardiac cycle, resulting in different time step sizes: 250 (3.69 ms), 500 (1.84 ms), 1000 (0.92 ms), and 2000 (0.46 ms). Note that the duration of the cardiac cycle was fixed, such that increasing the number of time steps, the time step size becomes smaller. The 500 time‐step configuration, which yields a time step size of about 1.84 ms, is the same as the time step used in the 1D model. Following the mesh analysis, where there was no difference between cycles for M3, all further simulations were conducted with two cardiac cycles. Time‐step dependence was assessed by the computation of the average inlet pressure in the second cycle, computed for the different time step resolutions. Figure B4 shows that the resulting pressure drop is largely unaffected by the choice of time step size. Only the simulation with 1000 timesteps per cycle deviates slightly, with a difference of just 0.9 mmHg in the area‐averaged pressure drop.

Additionally, we compared the COUPLED and SIMPLE pressure–velocity coupling approaches for a time step size of 1.84 ms. In SIMPLE, velocity and pressure are updated sequentially using a predictor–corrector method, whereas the COUPLED algorithm solves for both simultaneously, providing improved robustness and efficiency for both steady and transient flows under challenging conditions (i.e., poor mesh quality or large time steps). The comparison between the SIMPLE and COUPLED pressure–velocity coupling strategies shows that, in our case, the faster SIMPLE scheme produces results comparable to the COUPLED scheme, with a difference of $1\times 10^{-3}$ mmHg.

For all 3D simulations onwards, mesh sizes according to mesh M3 are chosen for all domains, with a time step size of 1.84 ms and a SIMPLE scheme.

## Appendix C Pressure Drop and Flow Waveforms Resulting From Transient Coupling Schemes

This section shows the resulting pressure and flow waveforms obtained from both the 1D and 3D simulations, for each different coupling method retrieved during the last cycle (Figures C2, C3, C4, C5, C6).
